# Sugarcane: an unexpected habitat for black yeasts in *Chaetothyriales*

**DOI:** 10.1186/s43008-023-00124-7

**Published:** 2023-10-04

**Authors:** Flávia de F. Costa, Rafael S. C. de Souza, Morgana F. Voidaleski, Renata R. Gomes, Guilherme F. Reis, Bruna J. F. de S. Lima, Giovanna Z. Candido, Marlon R. Geraldo, Jade M. B. Soares, Gabriela X. Schneider, Edvaldo da S. Trindade, Israel H. Bini, Leandro F. Moreno, Amanda Bombassaro, Flávio Queiroz-Telles, Roberto T. Raittz, Yu Quan, Paulo Arruda, Derlene Attili-Angelis, Sybren de Hoog, Vania A. Vicente

**Affiliations:** 1 Engineering Bioprocess and Biotechnology Post-Graduation Program, Department of Bioprocess Engineering and Biotechnology, Federal University of Paraná, Curitiba, Paraná, Brazil; 2 Molecular Biology and Genetics Engineering Center, State University of Campinas (UNICAMP), Campinas, São Paulo, Brazil; 3 Microbiology, Parasitology and Pathology Post-Graduation Program, Department of Basic Pathology, Federal University of Paraná, Curitiba, Paraná Brazil; 4 Biological Sciences Graduation, Federal University of Paraná, Curitiba, Paraná, Brazil; 5 Department of Cell Biology, Federal University of Paraná, Curitiba, Paraná, Brazil; 6 Clinical Hospital of the Federal University of Paraná, Federal University of Paraná, Curitiba, Paraná Brazil; 7 Laboratory of Bioinformatics, Professional and Technological Education Sector, Federal University of Paraná, Curitiba, Brazil; 8 Center of Expertise in Mycology of Radboud, University Medical Center / Canisius Wilhelmina Hospital, Nijmegen, The Netherlands; 9 Genetics and Evolution Department, Biology Institute, State University of Campinas (UNICAMP), Campinas, São Paulo, Brazil; 10 Division of Microbial Resources (DRM/CPQBA), State University of Campinas (UNICAMP), Campinas, São Paulo, Brazil

**Keywords:** Metagenomics, Selective isolation, *Cladophialophora bantiana*

## Abstract

Sugarcane (*Saccharum officinarum*, *Poaceae*) is cultivated on a large scale in (sub)tropical regions such as Brazil and has considerable economic value for sugar and biofuel production. The plant is a rich substrate for endo- and epiphytic fungi. Black yeasts in the family *Herpotrichiellaceae* (*Chaetothyriales*) are colonizers of human-dominated habitats, particularly those rich in toxins and hydrocarbon pollutants, and may cause severe infections in susceptible human hosts. The present study assessed the diversity of *Herpotrichiellaceae* associated with sugarcane, using in silico identification and selective isolation. Using metagenomics, we identified 5833 fungal sequences, while 639 black yeast-like isolates were recovered in vitro. In both strategies, the latter fungi were identified as members of the genera *Cladophialophora*, *Exophiala*, and *Rhinocladiella* (*Herpotrichiellaceae*), *Cyphellophora* (*Cyphellophoraceae*), and *Knufia* (*Trichomeriaceae*). In addition, we discovered new species of *Cladophialophora* and *Exophiala* from sugarcane and its rhizosphere. The first environmental isolation of *Cladophialophora bantiana* is particularly noteworthy, because this species up to now is exclusively known from the human host where it mostly causes fatal brain disease in otherwise healthy patients.

**Supplementary Information:**

The online version contains supplementary material available at 10.1186/s43008-023-00124-7.

## INTRODUCTION

Sugarcane (*Saccharum officinarum*, *Poaceae*) is a perennial grass cultivated on a large scale in tropical and subtropical regions (Lima et al. 2001). The economic interest in sugarcane is based on its main derivatives, which are sugar, alcohol, and bagasse, as well as a variety of by-products (Cheavegatti-Gianotto et al. 2011). In Brazil, sugarcane is an expanding crop covering the Midwest, Southeast, South, and Northeast regions (Cheavegatti-Gianotto et al. 2011; De Arruda et al. 2017). Sugarcane biomass is mainly composed of cellulose, hemicellulose, and lignin; sugar content is approximately 15.5 to 24% (Canilha et al. 2012). Upon decomposition in biotechnological processes, microbial degradation of lignin occurs through enzymes such as laccases and peroxidases (Kumar et al. 2009). Genomic studies of several black yeast-like fungi have identified the coding genes for these enzymes (Teixeira et al. 2017; Vicente et al. 2017; Moreno et al. 2017).

Black yeast-like fungi are melanized and belong to the orders *Dothideales* and *Chaetothyriales* (de Hoog et al. 2000). The most important family, *Herpotrichiellaceae* (*Chaetothyriales*) comprises environmental species (Vicente et al. 2014; Nascimento et al. 2017) of which a large number are able to cause severe opportunistic infections in humans (De Azevedo et al. 2015). Chromoblastomycosis, phaeohyphomycosis (Queiroz-Telles et al. 2017; Revankar et al. 2017), and primary brain infection (Horré & de Hoog 1999) are particularly significant. These etiologic agents have been reported from environmental samples and plants (Salgado et al. 2004; Vicente et al. 2008, 2014), leaf-cutting ants (Duarte et al. 2014), and particularly from domestic (Wang et al. 2018) and hydrocarbon-polluted (Isola et al. 2013) environments.

Clinical cases of chromoblastomycosis and phaeohyphomycosis are reported worldwide, and the World Health Organization (WHO) has classified the two diseases as Neglected Tropical Diseases (NTDs) (Queiroz-Telles et al. 2017; Revankar et al. 2017). Chromoblastomycosis is an occupational skin disease with acanthosis that affects rural workers exposed to soil and plant material. In Brazil, the highest incidence of chromoblastomycosis occurs in the Amazon region and Maranhão State, which are characterized as endemic areas (Gomes et al. 2016; Santos et al. 2020). Chromoblastomycosis is diagnosed by the presence of muriform fungal cells in expanding tissue. In contrast, phaeohyphomycosis leads to tissue invasion and necrosis by septate hyphae (Revankar et al. 2017; Arcobello & Revankar 2020). Severe, disseminated forms of these diseases are observed in patients with inherited *CARD9*-related immunodeficiency (Vaezi et al. 2018; Song et al. 2021).

The epidemiological data of these diseases suggest an environmental infection route, even though only few studies reported the presence of infectious species in the natural environment (Vicente et al. 2008, 2014; Salgado et al. 2004; Lima et al. 2020). Culture-independent methods provide an additional approach to environmental studies, due to their capacity to explore large areas revealing the presence of non-cultured species in complex samples by recognition of DNA markers (Cuadros-Orellana et al. 2013). Metagenomic data have successfully been applied in the family *Herpotrichiellaceae* in Brazilian sugarcane (Souza et al. 2016). Molecular markers were shown to be effective in demonstrating the environmental presence of agents of chromoblastomycosis and phaeohyphomycosis (Costa et al. 2020). The present study aims to analyze the prevalence of human-opportunistic members of the family *Herpotrichiellaceae* in sugarcane plants through combined in silico and isolation methods.

## MATERIALS AND METHODS

### Metagenomics dataset and molecular markers

Consulted metagenomic datasets of sugarcane are publicly available in the Sequence Read Archive (SRA) (https://www.ncbi.nlm.nih.gov/sra). The BioProject access number is PRJNA319259. The reads comprise the ITS2 region as described by Souza et al. (2016).

The sequences of molecular markers used for in silico identification of black yeasts were those of Najafzadeh et al. (2011, 2013, 2018), Hamzehei et al. (2013), Deng et al. (2014), Schneider et al. (2019), Feng et al. (2013), and Heinrichs et al. (2012) (Additional file 2: Table S1). A total of 71 species are represented by 106 barcodes (varying between 18 and 41 bp) and 42 padlock probe sequences (varying between 28 and 42 bp).

### Criteria for data mining

The metagenomic project was identified in the SRA database and our runs were indexed in web BLASTn (version 2.6.0. +). The comparison was performed through an archive multifasta containing the molecular marker sequences against the reads from the metagenome. Were considered only results with the following parameters as cutoff: alignments with coverage of 100% and identity of 100% (perfect match), identical to those described in previous studies (Costa et al. 2020).

### Study area and samples

A total of 11 environmental samples were analyzed, comprising five samples of living shoots of sugarcane, partitioned into leaf, stalk, and root, and six samples of soil rhizosphere. Samples were collected from two locations: (A) three shoots of sugarcane collected in Paulinia city, São Paulo State, Brazil (22°46′33.2"S, 47°05′55.7"W), and (B) two shoots of sugarcane and six samples of soil rhizosphere from the greenhouse in Campinas city, São Paulo State, Brazil (22°81′90.5"S, 47°05′92.8"W), being analyzed previously by Souza et al. (2016).

### Fungal isolation by oil flotation

Approximately 20 g sample per sugarcane plant (rhizosphere, root, stalk, and leaf) were subjected to oil flotation (Iwatsu et al. 1981; Vicente et al. 2008). Samples were incubated at room temperature for 30 min in 100 mL sterilized saline solution containing 200 U penicillin, 200 µg/L streptomycin, 200 µg/L chloramphenicol, and 500 µg/L cycloheximide. Subsequently, 20 mL of sterilized mineral oil was added, followed by vigorous shaking for 5 min. The flasks were left to settle for 20 min. Aliquots of the 100 µL oil–water interphase of each sample were carefully collected and inoculated onto Mycosel agar plates (Difco, Detroit, MI, U.S.A.) and incubated for 55 days at 28 °C, with a total of ten replicates per sample. Five plants were collected, separated into four samples, with ten replicates, leading to a total of 2000 replicates.

### Isolation of endophytic fungi

Isolation of endophytic fungi was conducted according to protocols of Petrini (1991) adapted by Lima (2008). Sugarcane parts were divided into four fragments (approximately 0.5 cm^2^) by a flame-sterilized blade in a laminar flow hood to prevent contamination by spores from the air. In addition, surfaces were sterilized by immersion in 70% ethanol for 1 min, sodium hypochlorite 124 (2–2.5% active chlorine) for 4 min, 70% ethanol for 30 s and washed thrice with sterile distilled water. The fragments were transferred aseptically to Mycosel agar (Difco, Detroit, MI) and incubated for 55 days at 28 °C.

### Morphological identification

A preselection of black yeast-like isolates was done, strains being transferred to Sabouraud’s glucose agar (SGA) at room temperature and evaluated by slide culture (de Hoog et al. 2011; Vicente et al. 2014). The fungi were inoculated onto Oatmeal agar blocks, covered with sterilized glass slides, and incubated at 28 °C for 7, 14 and 21 days. The micromorphology was used for preliminary identification and attribution to main groups.

### DNA extraction

DNA extraction was done according to Vicente et al. (2008). Fungal material was macerated in a microtube containing silica:celite (2:1), 200 μL CTAB and 500 μL CIA (acidic chloroform isoamyl alcohol solution) with centrifugation for 7 min. to 16,000 g. DNA was precipitated with 96% ice-cold alcohol, followed by two washes with 500 μL 70% ethanol, dehydrated and again hydrated with ultrapure water. DNA was quantified using NanoDrop (2000®, Thermo Scientific, Waltham, MA) spectrophotometer and integrity was checked by 0.8% agarose gel electrophoresis.

### Molecular identification

The initial identification of the isolates was based on sequencing of the rDNA Internal Transcribed Spacer (ITS) rDNA region. The taxonomic position of the isolates was confirmed by additional sequencing of the partial large subunit of the nuclear ribosomal DNA gene (LSU), β-tubulin (*BT2*), and translation elongation factor 1-α (*TEF1*). The primers used for the amplification are listed in Table 1. PCR reactions were performed in a 12.5 μL volume of a reaction mixture containing 1 × PCR buffer, 2.0 mM MgCl_2_, 25 μM dNTPs, 0.5 μM of each forward and reverse primer, 1 U DNA polymerase (Ludwig Biotec, Bela Vista, Brazil) and 20 ng genomic DNA. Amplification was performed in an ABI Prism 2720 thermocycler (Applied Biosystems, Foster City, USA) as follows: 95 °C for 5 min, followed by 35 cycles consisting of 94 °C for 45 s (denaturation), 52 °C for 45 s (annealing), and 72 °C for 2 min (extension), with a final delay step at 72 °C for 7 min, for LSU and ITS. For *BT2,* the annealing temperature was changed to 58 °C and for *TEF1* to 56 °C. Amplicons were cleaned with Exonuclease I and Shrimp Alkaline Phosphatase (SAP) according to manufacturer’s instructions. The same primers presented in Table 1 were used for sequencing reactions applying the Big Dye terminator cycle sequencing RR mix protocol (ABI PRISM v3.1, Applied Biosystems, Foster City, CA) with the following conditions: 96 °C for 2 min, 96 °C for 10 s, 52 °C for 10 s, 62 °C for 4 s, with 35 cycles. Products were purified using Sephadex G-50 fine (GE Healthcare Bio Sciences, Uppsala, Sweden).

**Table 1:** Primers and PCR conditions used in DNA sequencing

Gene	Primers	Oligonucleotides (5′-3′)	References
Large subunit ribosomal DNA (LSU)	NL1	GCATATCAATAAGCGGAGGAAAAG	(O'Donnell 1992; Vilgalys & Hester 1990)
	LR5	TCCTGAGGGAAACTTCG	
Internal Transcribed Spacer rDNA (ITS)	ITS1	TCCGTAGGTGAACCTGCGG	(White et al. 1990)
	ITS4	TCCTCCGCTTATTGATATGC	
β-tubulin (*BT2*)	Bt-2a	GGTAACCAAATCGGTGCTGCTTTC	(Glass & Donaldson 1995)
	Bt-2b	ACCCTCAGTGTAGTGACCCTTGGC	
Translation Elongation factor 1-α (*TEF1*)	EF1-728F	CATCGAGAAGTTCGAGAAGG	(Carbone & Kohn 1999)
	EF1-986R	TACTTGAAGGAACCCTTACC	

### Phylogenetic analysis

Consensus sequences of the ITS, *BT2*, *TEF1* and the LSU regions were adjusted using the BioEdit Sequence Alignment Editor v7.2.5 (Hall 1999) and alignments of obtained sequences were performed using MAFFT (Katoh et al. 2018). The isolates provisionally identified by morphology were first identified based on ITS rDNA sequences by comparison with reference sequences available in GenBank (Additional file 3: Table S2) and in an in-house ribosomal alignment containing types of all described species (26 June 2023). Sequence comparisons were performed in the UNITE database (https://unite.ut.ee/). Sequences with homology greater than or equal to 99% identity with type strains were considered as correctly identified. The LSU region was used to reconstruct the phylogeny of the *Herpotrichiellaceae* showing approximate groups that were recognized previously (Quan et al. 2020; de Hoog et al. 2011; Teixeira et al. 2017). Separate trees based on LSU sequences were built with 1000 bootstrap replicates using Maximum Likelihood implemented in MEGA v7 software (Kumar et al. 2016) and Bayesian inference (BI) in MrBayes v3.2.6. Trees were visualized in FigTree v1.4.3 (http://tree.bio.ed.ac.uk/software/Fig.tree/)

Multiple sequence alignments were made by MAFFT v7 (http://mafft.cbrc.jp/) and optimized manually using MEGA v7.2 (Kumar et al. 2016). For species delimitation, initially each region of ITS, *TEF1* and *BT2* was analyzed separately using maximum likelihood (ML) algorithm. Subsequent analysis was performed with combined data from three gene regions by ML implemented in MEGA v7.0.26 with the Tamura-Nei model.

The sequences were deposited in GenBank and compared with related reference strains (Additional file 3: Table S2). The holotype numbers were provided with the deposit of the fungal exsiccates at the Department of Botany Herbarium at the Federal University of Paraná (UPCB) with the accredited records on the MycoBank Database. The isolated strains were deposited in The Microbiological Collections of Paraná Network—CMRP/Taxonline (https://www.cmrp-taxonline.com/), preserved with long-term conservation methods, including cryopreservation and DNA storage. Data are registered in the database and available at the SpeciesLink network (https://www.cmrp-taxonline.com/catalogue).

### Physiology

Cardinal growth temperatures were determined on SGA after incubation for 3 weeks at 18–42 °C with 3 °C increments (Vicente et al. 2014). All tests were performed in triplicate and the diameters of the colonies were recorded in the last week. Experiments consisted of three simultaneous replicates for each tested strain; averages of three measurements were calculated. Growth velocities per species were obtained by calculation of the average values and the respective standard deviations. Results were plotted with temperature (°C) *versus* colony diameter (mm) as parameters. Optimum range (= average ± standard deviation) and maximum growth temperatures among species were determined with three replicates.

Tests for *Cladophialophora bantiana* (CMRP3443) were performed according to de Hoog et al. (1995). The carbon sources (D-glucose, D-ribose, L-arabinose, D-arabinose, L-rhamnose, sucrose, maltose, melibiose, lactose, soluble starch, glycerol, meso-erythritol and D-mannitol) were weighed (1.68 g) and dissolved in 100 mL yeast nitrogen base medium (Difco, Detroit, MI, U.S.A.), distributed in volumes of 4.5 mL in tubes and sterilized by filtration over 0.22 µm pore-size. Ethanol was added as 3 drops (30 µL) after sterilization of Yeast nitrogen base medium. The growth at carbon sources that showed the highest assimilation (glucose, sucrose, and soluble starch) were verified in concentrations of 5–60% of the carbon source using the same ratio of yeast nitrogen base medium as for carbon assimilation tests. For nitrogen assimilation tests, the carbon sources were prepared with 11.7 g Yeast carbon base medium (Difco, Detroit, MI) in 100 mL distilled water. Solutions were filtered using a 0.22 µm Millipore filter. Nitrogen assimilation tests were done with 0.78 g potassium nitrate, 0.26 g sodium nitrate and 0.56 g L-lysine. For halotolerance tests, 7.5 g and 15 g (NaCl) and magnesium chloride (MgCl_2_) were weighed and dissolved separately in 150 mL glucose solution, filter-sterilized, and distributed aseptically in 4.5 mL volumes in tubes previously sterilized for 60 min at 1 ATM pressure. Osmophily was determined with sugar tolerance in a 5–60% final concentration range for glucose and sucrose in Yeast nitrogen base medium. Fungal inocula of 0.5 mL were prepared as suspensions of 10^7^ conidia/mL in Yeast nitrogen base medium (Difco, Detroit, MI). Tubes were incubated at 36 ºC in an upright position, shaken at 150 rpm. The negative control was the medium without cells. Fermentation of carbon sources was verified after incubation for two weeks in vertical tubes with Durham inserts, with fermentation basal medium (0.45% powdered yeast extract and 0.75% peptone). Biomass production was evaluated visually by turbidity after two weeks. The ( +) denominates positive growth, (-) indicates absence of growth and (w) weak growth compared to the positive control.

## RESULTS

**Fig. 1:**
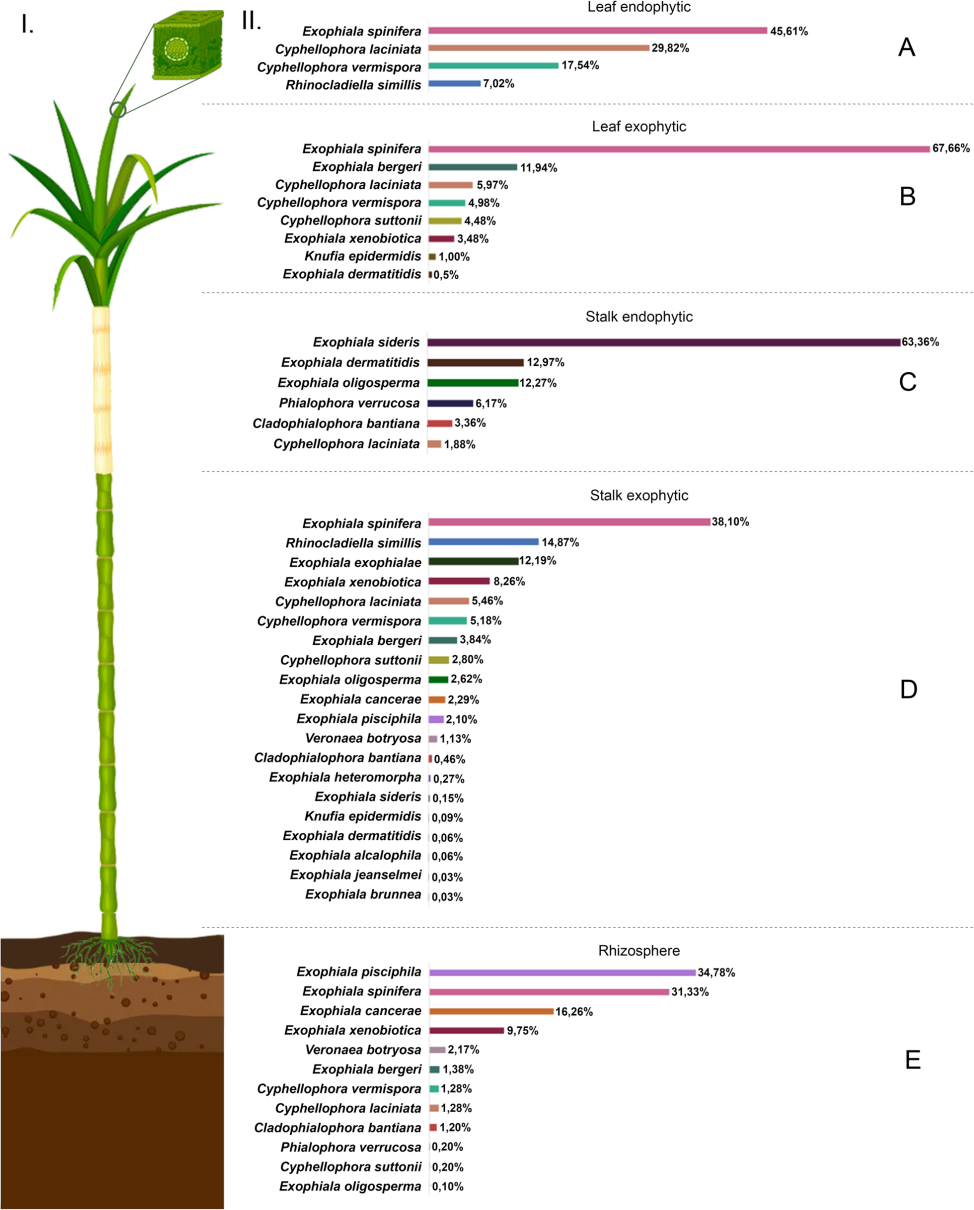
Distribution of the fungi in *Herpotrichiellaceae* on sugarcane, based on the specific molecular markers applied in the metagenome database. I. Evaluated metagenomic samples from different parts of sugarcanes from São Paulo, Brazil. II. Percentage of metagenomic sequences from BioProject PRJNA319259: **A** in leaf endophytic, **B** in leaf exophytic, **C** in stalk endophytic, **D** in stalk exophytic, and **E** rhizosphere samples

From the consulted metagenomic datasets of fungi associated to sugarcane that were publicly available, a total of 5,833 sequences related to chaetothyrialean black yeasts were identified, from the rhizosphere (1014 sequences), leaf endophytic (57 sequences), leaf exophytic (201 sequences), stalk endophytic (1,280 sequences) and stalk exophytic (3,281 sequences). In the analysis, 21 species were identified, i.e., *Cladophialophora bantiana*, *Cyphellophora laciniata*, *Cy. suttonii*, *Cy. vermispora*, *Exophiala alcalophila*, *E. bergeri*, *E. brunnea*, *E. cancerae*, *E. dermatitidis*, *E. exophialae*, *E. heteromorpha*, *E. jeanselmei*, *E. oligosperma*, *E. pisciphila*, *E. sideris*, *E. spinifera*, *E. xenobiotica*, *Knufia epidermidis*, *Phialophora verrucosa*, *Rhinocladiella similis,* and *Veronaea botryosa*, distributed throughout the sugarcane organs (Fig. 1, Additional file 4: Table S3).

The new taxa obtained as cultures by isolation from sugarcane were later identified in all sugar plant samples (rhizosphere, root, stalk, and leaf) by analysis in silico in the same database analyzed in this study.

Selective isolation by oil flotation yielded 639 cultures of black yeast-like fungi from 10 replicates per sample, with a total of 250 replicates. Forty-two isolates were selected for molecular identification based on morphological characteristics shared with agents of chromoblastomycosis and phaeohyphomycosis. Colonies of black yeast-like fungi were obtained from all sugarcane fragments (Table 2), although a higher number of isolates (*n* = 591) was recovered by selective isolation than by the endophytic method (*n* = 48).

**Table 2:** Percentages of numbers of isolates of Black yeast-like fungi (100% = 639 cultures) obtained from different parts of sugarcanes growing in São Paulo, Brazil

Parts of sugarcane	Isolation methods			% Isolation per sample	Total of isolates
	Oil flotation		Endophytic		
	Samples A	Samples B	Samples A		
Rhizosphere	11	24	–	5.47	35
Root	192	1	32	35.21	225
Stalk (surface)	17	200	–	33.95	217
Stalk (inside)	–	37	16	8.29	53
Leaf	109	0	0	17.05	109
Total	329	262	48	–	639

Samples A (Paulinia city); Samples B (Campinas city)

**Fig. 2:**
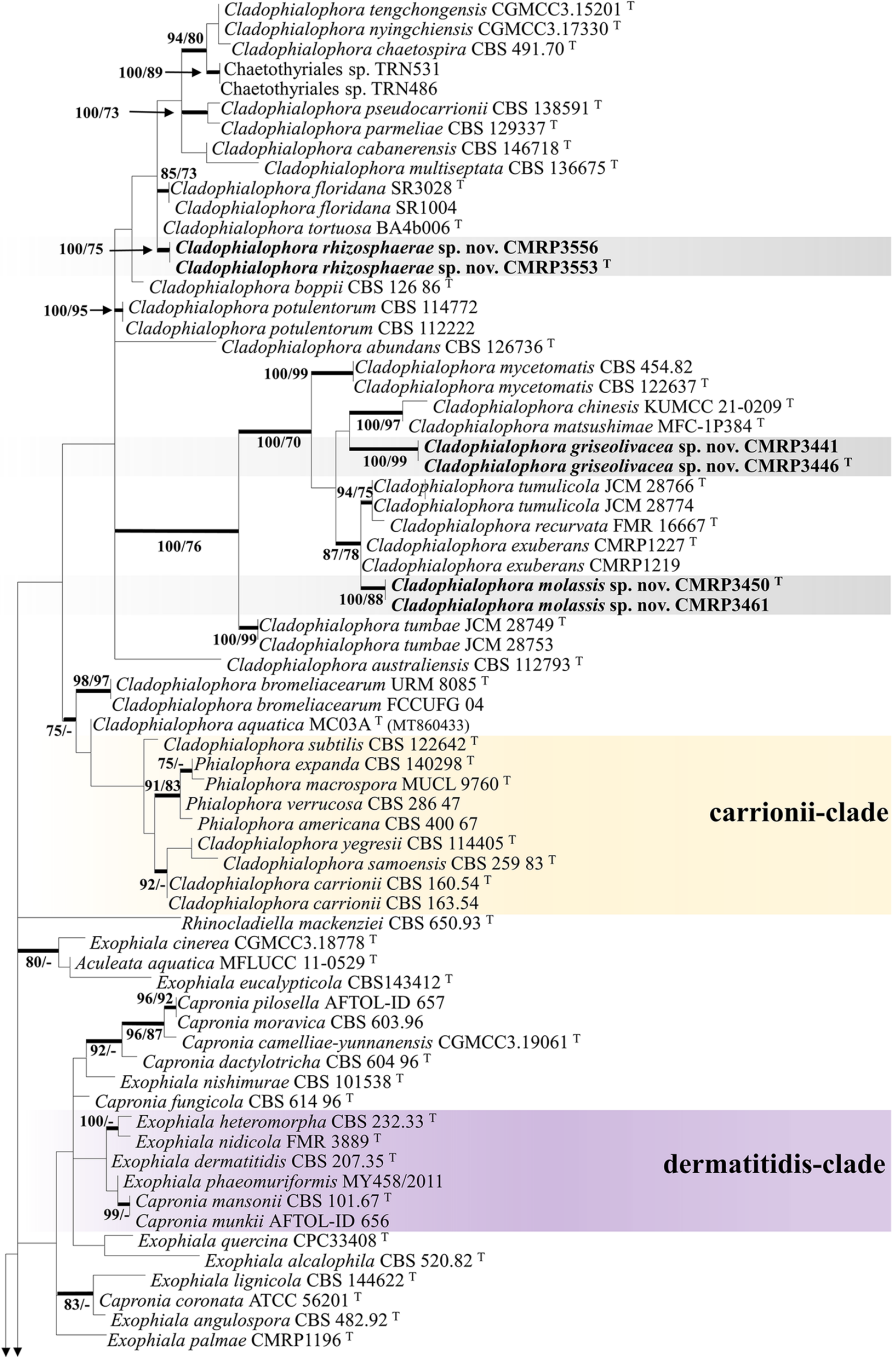

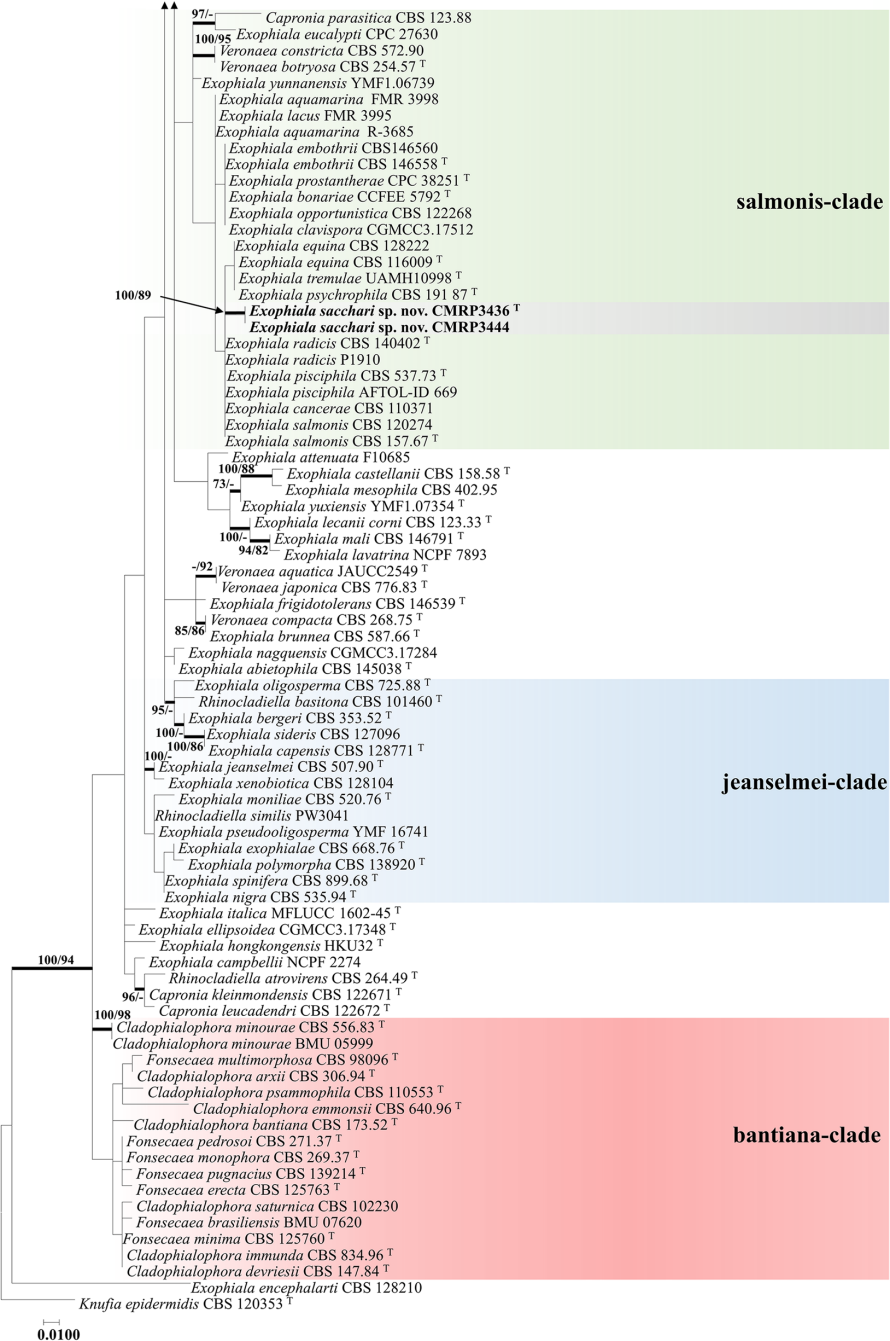
Phylogeny of a representative selection of species in *Chaetothyriales* based on LSU rDNA sequences, constructed by Bayesian analysis and maximum likelihood. Values of > 70% for Bayesian probability (left) and Bootstrap values > 70% for maximum likelihood (right) from 1000 resampled datasets are shown with branches. Novel species are indicated in bold. The outgroup was *Knufia epidermidis* (CBS 120353). ^T^ = Ex-type strain

**Fig. 3:**
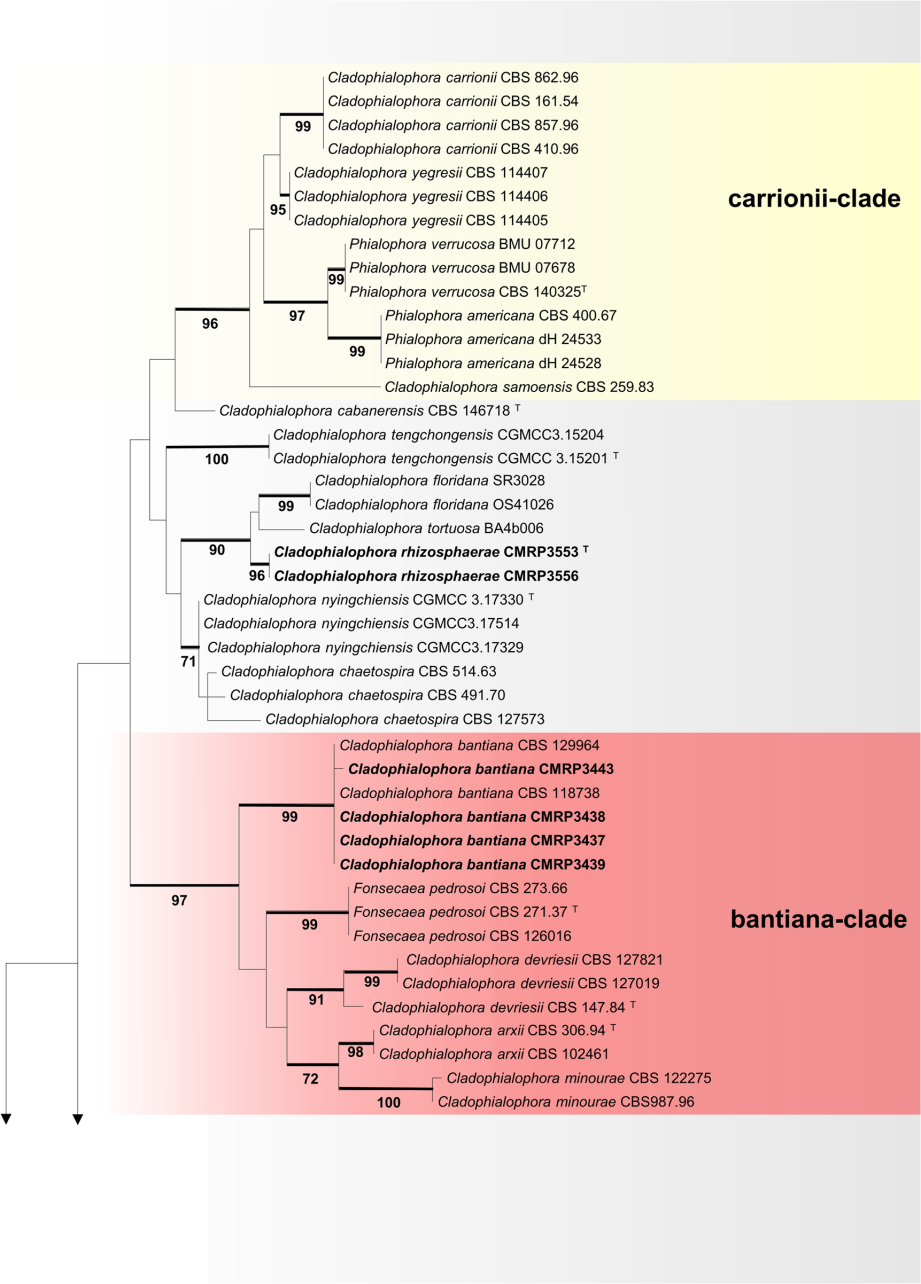

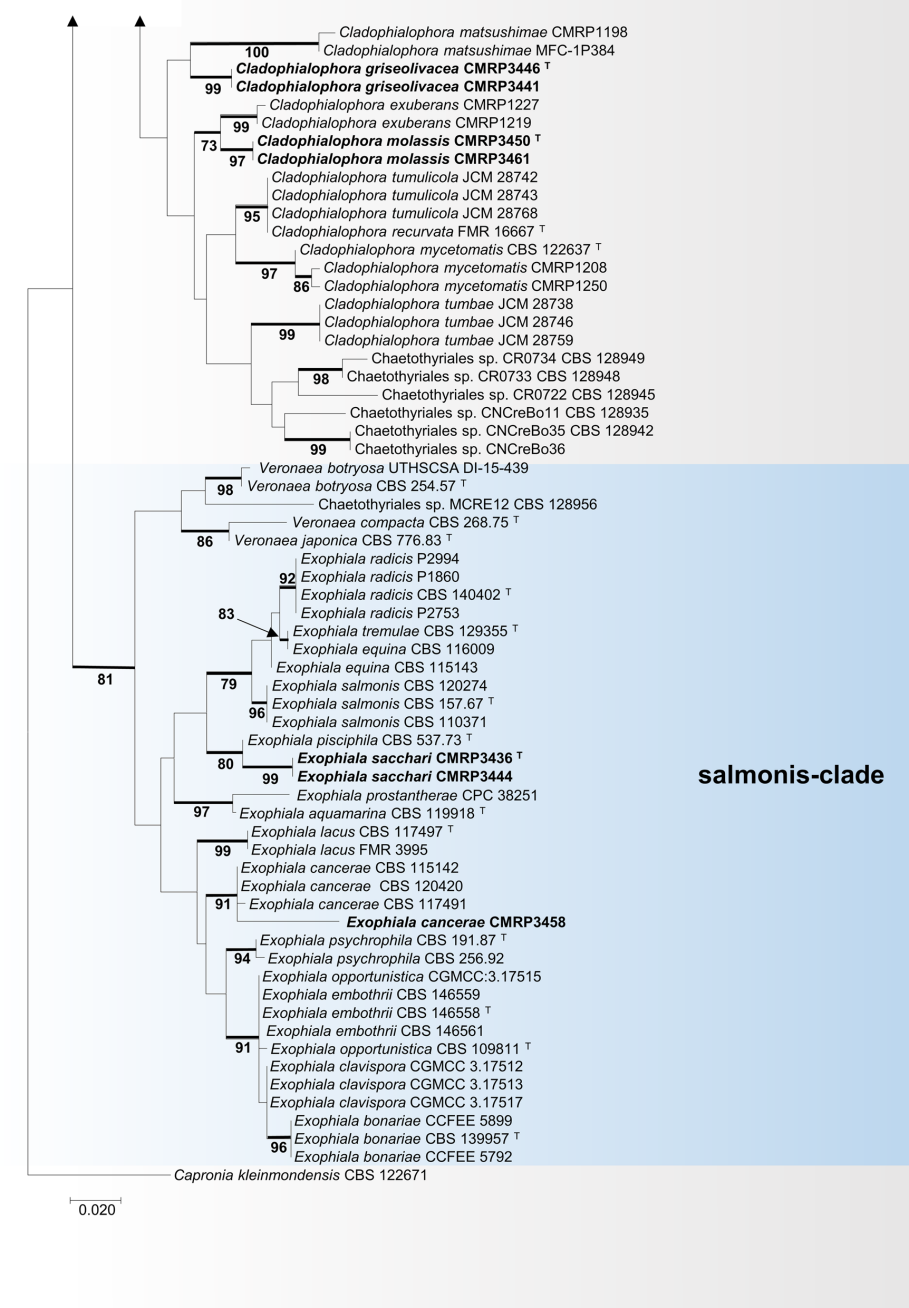
Phylogenetic tree of *Herpotrichiellaceae* based on the alignment of ITS1-5.8S-ITS2 and β-tubulin sequences, constructed with Maximum likelihood implemented in MEGA7 with Tamura-Nei model. *Capronia kleinmondensis* (CBS 122671) was chosen as outgroup. Bootstrap values (1000 replicates) above 70% are added to supported branches. Novel species and isolates of *Cladophialophora bantiana* and *Exophiala cancerae* are indicated in bold. ^T^ = Ex-type strain

The sugarcane samples yielded isolates of a diversity of species of black yeasts (Table 3), among which were *Cladophialophora bantiana* (4 isolates), *C. floridana* (2), *Cyphellophora oxyspora* (1), *Exophiala cancerae* (1), *E. lecanii-corni* (2), *E. spinifera* (2), and *Rhinocladiella similis* (6) (Additional file 1: Fig. S1). In addition, unidentifiable strains were recovered as *Cladophialophora* sp. (21) and *Exophiala* sp. (2). In order to assess the taxonomic position of new isolates that did not match with any described taxon, a tree of partial LSU rDNA (Fig. 2) was made. Judging from the LSU analysis, the cladophialophora-like isolates clustered with *Cladophialophora* species in different clades, while *Exophiala* spp. isolates were located at the salmonis-clade amidst waterborne *Exophiala* species (Fig. 2). Two isolates of each new species were selected for the analysis of sequences of further gene regions and to build a multilocus tree based on ITS, *BT2* and *TEF1* (Fig. 3). The trees were constructed using sequence data of representative species in *Herpotrichiellaceae* (*Chaetothyriales*), based on Quan et al. (2020). All reference strains used in the phylogenetic analysis are presented as in Additional file 3: Table S2.

**Table 3:** Molecular identification of environmental isolates from sugarcane growing in São Paulo, Brazil

Species	Strain	Isolation	Source	GenBank (ITS, LSU, *BT2*, *TEF1*)
*Chaetothyriales* sp.	CMRP3453	Oil flotation	Root	MW852485, -, -, -
*Cladophialophora bantiana*	CMRP3443	Oil flotation	Rhizosphere	MW656217, -, ON494575, -
	CMRP3437	Oil flotation	Rhizosphere	MW657823, -, ON494576, -
	CMRP3438	Oil flotation	Rhizosphere	MW656223, -, ON494577, -
	CMRP3439	Oil flotation	Rhizosphere	MW657363, -, ON494578, -
*Cladophialophora floridana*	CMRP3574	Oil flotation	Root	MW861542, -, -, -
	CMRP3579	Oil flotation	Root	MW861544, -, -, -
***Cladophialophora griseolivacea***	**CMRP3446**^**T**^	Oil flotation	Root	MZ048747, MW861546, ON553224, OQ348498
	CMRP3441	Oil flotation	Root	MZ029088, MW861545, ON553225, OQ348499
	CMRP3518	Oil flotation	Root	MZ052078, -, -, -
	CMRP3520	Oil flotation	Root	MZ052075, -, -, -
	CMRP3449	Oil flotation	Root	MZ052080, -, -, -
	CMRP3566	Oil flotation	Root	MZ052079, -, -, -
	CMRP3567	Oil flotation	Root	MZ052074, -, -, -
	CMRP3456	Oil flotation	Stalk	MZ052076, -, -, -
	CMRP3572	Oil flotation	Stalk	MZ052077, -, -, -
***Cladophialophora molassis***	**CMRP3450**^**T**^	Oil flotation	Leaf	MZ132103, MW865735, ON455204, OQ348500
	CMRP3536	Oil flotation	Leaf	MZ132098, -, -, -
	CMRP3676	Oil flotation	Leaf	MZ132097, -, -, -
	CMRP3680	Oil flotation	Leaf	MZ132095, -, -, -
	CMRP3565	Oil flotation	Root	MZ132102, -, -, -
	CMRP3716	Oil flotation	Root	MZ132099, -, -, -
	CMRP3525	Oil flotation	Root	MZ132096, -, -, -
	CMRP3461	Oil flotation	Root	MZ126811, MW865734, ON455205, OQ348501
	CMRP3485	Endophytic	Endophytic root	MZ132101, -, -, -
	CMRP3559	Endophytic	Endophytic root	MZ132094, -, -, -
***Cladophialophora rhizosphaerae***	**CMRP3553**^**T**^	Oil flotation	Rhizosphere	MZ006214, MW856019, ON553222, OQ348496
	CMRP3556	Oil flotation	Rhizosphere	MZ008436, MW715827, ON553223, OQ348497
*Cyphellophora oxyspora*	CMRP3526	Oil flotation	Rhizosphere	MT331614, -, -, -
*Exophiala cancerae*	CMRP3458	Oil flotation	Root	MW817562, -, -, -
*Exophiala lecanii-corni*	CMRP3664	Oil flotation	Root	MT448881, -, -, -
	CMRP3747	Oil flotation	Root	MT452654, -, -, -
***Exophiala sacchari***	**CMRP3436**^**T**^	Oil flotation	Rhizosphere	MZ132100, MW881154, ON455203, OQ348494
	CMRP3444	Oil flotation	Rhizosphere	MZ130934, MW881155, ON454893, OQ348495
*Exophiala spinifera*	CMRP3442	Endophytic	Endophytic root	MT448889, -, -, -
	CMRP3538	Oil flotation	Root	MT452653, -, -, -
*Rhinocladiella similis*	CMRP3524	Oil flotation	Root	MK603867, -, -, -
	CMRP3523	Oil flotation	Root	MT322620, -, -, -
	CMRP3448	Endophytic	Endophytic stalk	MK645320, -, -, -
	CMRP3454	Oil flotation	Leaf	MK645599, -, -, -
	CMRP3457	Oil flotation	Leaf	MT322621, -, -, -
	CMRP3675	Oil flotation	Leaf	MW656170, -, -, -

Novel species and ex-type strains are indicated in bold, and ex-type strains also by “^T^”

A multilocus tree based on ITS, *TEF1,* and *BT2* sequences was built with Maximum Likelihood implemented in RaxML v7.0.4 using the General time reversible substitution model. A total of 1,326 sites were evaluated for ITS, *BT2*, and *TEF1*, corresponding to 630, 458, and 238 sites of the respective genes. Of these, 1,326 were conserved, 743 were variable, 661 were parsimony informative (pi), and 36 were unique. The empirical base frequencies were 0.23070 for pi(A), 0.23823 for pi(C), 0.24061 for pi(G), and 0.24637 for pi(T), with 1,000 bootstrap inferences. *Capronia kleinmondensis* was selected as outgroup. Results of *BT2* and ITS sequencing revealed a single environmental isolate of *Exophiala cancerae* and four of *Cladophialophora bantiana* among the sugarcane root and rhizosphere isolates, respectively (Fig. 3).

The phylogenetic analysis (Fig. 3) revealed that the environmental strains CMRP3553 and CMRP3556 of *Cladophialophora* grouped in a distinct cluster close to the type of strain *C*. *tortuosa* (BA4b006) that originates from sclerotia of the fungus *Cenococcum* sp., while strains CMRP3446 and CMRP3441 of *Cladophialophora* formed a separate cluster closely related to *C. mycetomatis*, a species from a human subcutaneous infection. In addition, the *Cladophialophora* strains CMRP3461 and CMRP3450 formed a separated group, close to *C*. *exuberans* from a decaying coconut shell*.* Judging from this analysis, the isolates of *Cladophialophora* are separate from previously described taxa (Figs. 2 and 3) and three of them will be introduced below as new species, namely *C. rhizosphaerae, C. griseolivacea* and *C. molassis*. Similarly, the *Exophiala* isolates CMRP3444 and CMRP3436 are close to *E. pisciphila* but at significant distance from known *Exophiala* species in the Salmonis clade (Fig. 3). Therefore, a novel taxon in *Exophiala* is introduced here, namely *Exophiala sacchari*.

## TAXONOMY

**Fig. 4:**
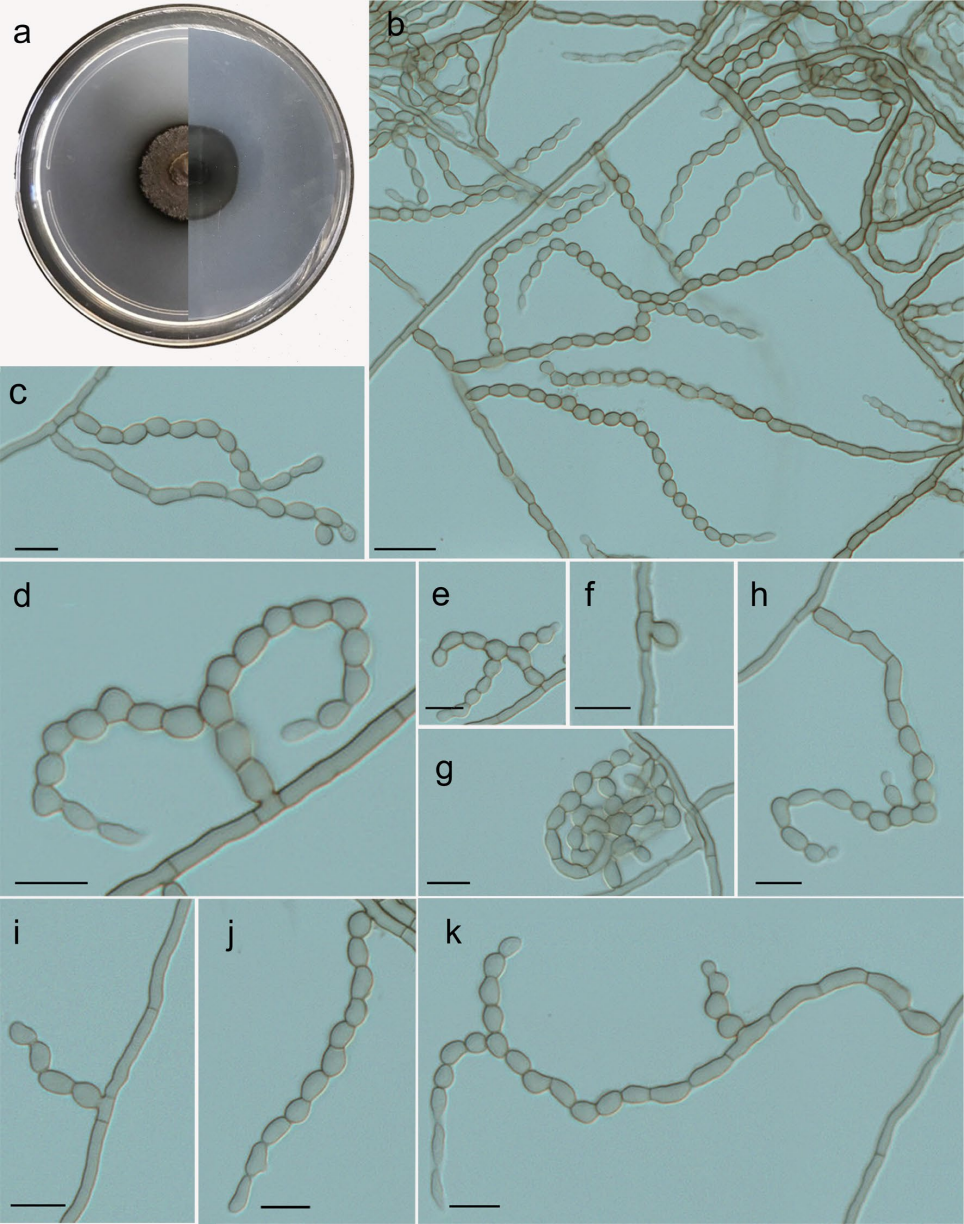
*Cladophialophora rhizosphaerae* microscopic morphology (UPCB 96826). **A** Colony on MEA; **B–C** Long conidial chains; **D–E** Conidial chains arranged sinuously; **G** Conidial clusters; **H** Coherent conidial chain with budding cells; **I–K** Conidial chains with ellipsoid to ovoid conidia. Bars = 10 μm

***Cladophialophora rhizosphaerae*** Costa, de Hoog, Gomes & Vicente, **sp. nov.** (Fig. 4).

MycoBank no.: MB839692.

*Etymology:* The name refers to the rhizosphere of sugarcane from which the isolates were recovered.

*Diagnosis*: The species differs from its nearest neighbours *C*. *tortuosa* and *C*. *floridana* sequences of ITS (96/95%), and LSU (99/98%) respectively.

*Type:* **Brazil:** *São Paulo state*: Campinas city, greenhouse, 22.81926^o^ W, 47.05930° S, isolated from the rhizosphere of sugarcane (*Saccharum officinarum*), (5 Apr. 2018, *F.F. Costa* (UPCB 96826 – holotype [dried culture]; CMRP3553 – ex-type culture. Sequences in GenBank: ITS MZ006214.1, LSU MW856019.1, *BT2* ON553222, *TEF1* OQ348496.

*Description: Macromorphology:* Colonies on SGA and MEA medium after 2 wk incubation at 28 ºC growing slowly, 18 − 19 mm diam. Colonies moderately expanding, greyish olive superficially, dark olive in agar, with olivaceous black reverse. Colonies circular, velvety with floccose grey mycelium, moist and slimy at the margin, with some pigment diffusing into the agar. *Micromorphology: Hyphae* mid- to pale olivaceous brown, 2 μm wide, septate every 7–19 μm, forming long acropetal conidial chains that are mostly unbranched and arise terminally or laterally, eventually arising in clusters. Budding cells are occasionally present. *Conidiogenous cells* undifferentiated. *Conidia* ellipsoidal to lemon-shaped, 4.5–8.2 × 2.5–4 μm diam, showing average size of conidia 6.7 × 3.2 μm diam.

*Notes***:** *Cladophialophora rhizosphaerae* is related to *C*. *tortuosa* and *C*. *floridana* in our phylogenetic analyses (Figs. 2 and 3). However, these species present some differences in morphology. The conidia and apical cells of the conidiophores are usually distinctly bent in *C*. *tortuosa,* whereas these cells are more or less straight in *C*. *floridana* (Obase et al*.* 2015). *Cladophialophora rhizosphaerae* forms long acropetal conidial chains that are mostly unbranched and arise terminally or laterally. Based on a BLASTn search in the GenBank nucleotide database, the closest hits using the ITS sequence was with *C*. *tortuosa* [strain BA4b006, GenBank AB986424.1; identities = 498/517 (96%), 3 gaps (0%)], *C*. *floridana* [(strain SR1004, GenBank AB986344.2; identities = 514/540 (95%), 8 gaps (1%)].

Additional material examined is listed in Table 3.

**Fig. 5:**
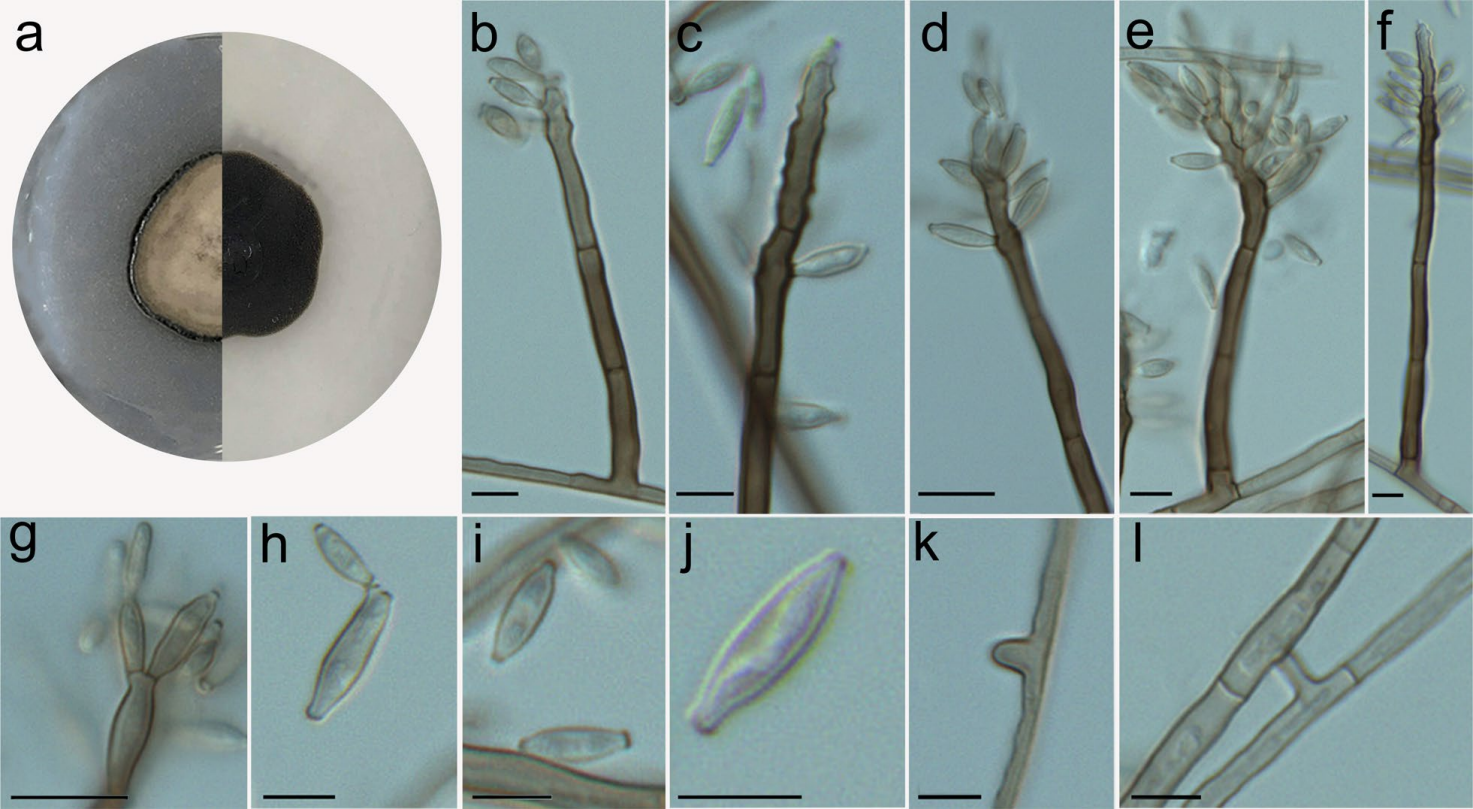
*Cladophialophora griseolivacea,* microscopic morphology (UPCB 96824). **A** Colony on SGA; **B–G** Conidiophores with conidia produced sympodially; **E–F** Proliferating rachis on conidiogenous cells, presenting slightly prominent and unpigmented scars; **H–J** Conidia; **K** Hyphae with chlamydospore-like cells; **L** Anastomosis. Bars = 10 μm

***Cladophialophora griseolivacea*** Costa, de Hoog, Gomes & Vicente, **sp. nov.** (Fig. 5).

MycoBank no.: MB839693.

*Etymology:* The name refers to the colour of the colony of this species.

*Diagnosis*: The species differs from its nearest neighbour *C*. *matsushimae* sequences of ITS (89%), and LSU (97%).

*Type:* **Brazil:** *São Paulo state*: Paulinia city, 22.77584° W,47.09883 ^o^ S isolated from sugarcane plant (*Saccharum officinarum*, (5 Apr. 2018, *F.F. Costa* (UPCB 96824 – holotype [dried culture]; CMRP3446 – ex-type culture). Sequences in GenBank: ITS (MZ048747.1), LSU (MW861546.1), *BT2* (ON553224), *TEF1* (OQ348498).

*Description: Macromorphology: Colonies* on SGA or MEA medium after 2 wk incubation at 28 ºC moderately expanding, 18–19 mm diam. Colonies greyish olive with olivaceous black reverse. Colonies restricted, circular, grey, later (at 14d) becoming brownish grey, velvety at the centre and moist and slimy at the margin. *Micromorphology: Hyphae* pale olivaceous to brown, 1.5 μm wide, septate every 18.7–26.3 μm, with dark brown differentiated, thick-walled (to 0.25 µm) conidiophores measuring 13–20 × 3–4 μm, presenting slightly prominent denticles. *Conidia* fusiform, pale to olivaceous, with discernible scars, 3.8–8.4 × 1.1–2.4 μm, average 5.2 × 1.7 μm (Fig. 5).

*Notes*: *Cladophialophora griseolivacea* is related to *C*. *matsushimae* in our phylogenetic analyses (Figs. 2 and 3). However, the species present differences in morphology, conidia being septate and more variable in shape in *C*. *matsushimae.* (Kuokol 2009). In *C. griseolivacea,* conidia are consistently fusiform. Based on a BLASTn search in the GenBank nucleotide database, the closest hits using the ITS sequence were with *C*. *matsushimae* [strain MFC-1P384, GenBank FN549916.1; identities = 454/509 (89%), 11 gaps (2%)].

Additional material examined is listed in Table 3.

**Fig. 6:**
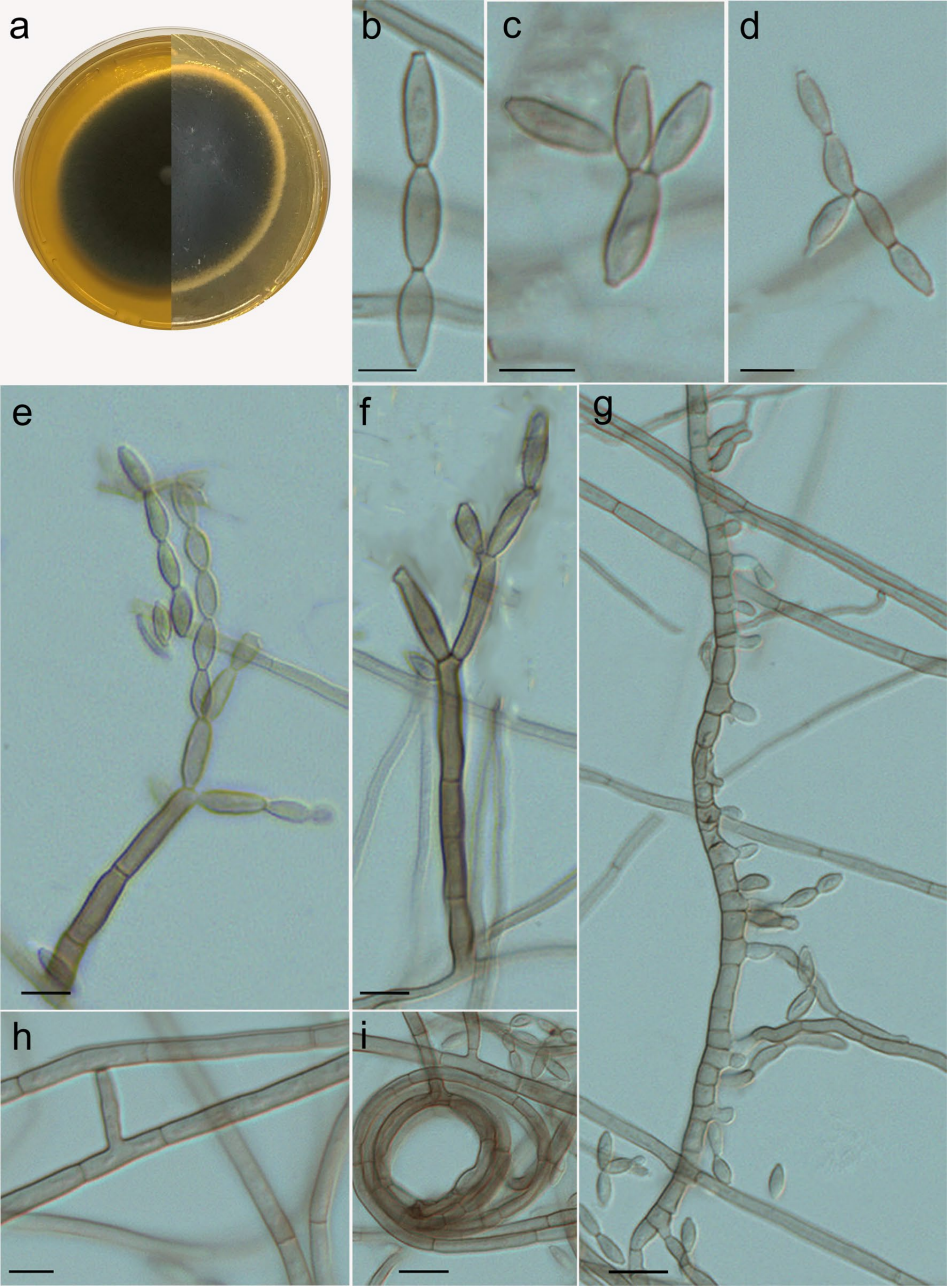
*Cladophialophora molassis* microscopic morphology (UPCB 96823). **A** Colony on MEA; **B-D** Conidia; **E** Fertile hyphae; **F** Conidiophore; **G** Hyphae; **H** Anastomosis; **I** Spirally twisted hyphae. Bars = 10 μm

***Cladophialophora molassis*** Costa, de Hoog, Gomes & Vicente, **sp. nov.** (Fig. 6).

MycoBank no.: MB839694.

*Etymology:* The name refers to a subproduct of the sugarcane, the molasses.

*Diagnosis*: The species differs from its nearest neighbour *C*. *exuberans* sequences of ITS (96%), and LSU (99%).

*Type:* **Brazil:** *São Paulo state*: Paulinia city, 22.77584° W,47.09883 ^o^ S, isolated from sugarcane plant (*Saccharum officinarum*, 5 Apr. 2018, *F.F. Costa* (UPCB 96823– holotype [dried culture]; CMRP3450 – ex-type culture). Sequences in GenBank: ITS (MZ132103.1), LSU (MW865735.1), *BT2* (ON455204), *TEF1* (OQ348500).

*Description: Macromorphology: Colonies* on SGA medium after 2 wk incubation at 28 ºC moderately expanding, circular, powdery, with a black center and greyish flat margin. Reverse olivaceous black, without diffusible pigment. *Micromorphology:* Fertile hyphae pale olivaceous brown, 1.9–3.2 μm wide, septate every 16–38 μm, forming long acropetal, branched or unbranched conidial chains. Erect conidiophores eventually present. Hyphae with septation every 6.5–11.3 μm. Spirally twisted hyphae and anastomosis eventually present. *Conidia* pale brown, lemon-shaped to fusiform with dark scars, one-celled, 3.8–6.9 × 1.4–2.6 μm, average 4.5 × 1.9 μm (Fig. 6).

*Note***:** *Cladophialophora molassis* is related to *C*. *exuberans* in our phylogenetic analyses (Figs. 2 and 3). However, the species present differences in morphology, Conidia are ellipsoidal, produced in long chains in *C*. *exuberans* (Nascimento et al. 2017), while in *C. molassis* they are lemon-shaped to fusiform arranged in small chains. Based on a BLASTn search of GenBank nucleotide database, the highest ITS similarity was with *C*. *exuberans* [strain CMRP1204, GenBank KY680432.1; identities = 492/510 (96%), 3 gaps (0%)].

Additional material examined is listed in Table 3.

**Fig. 7:**
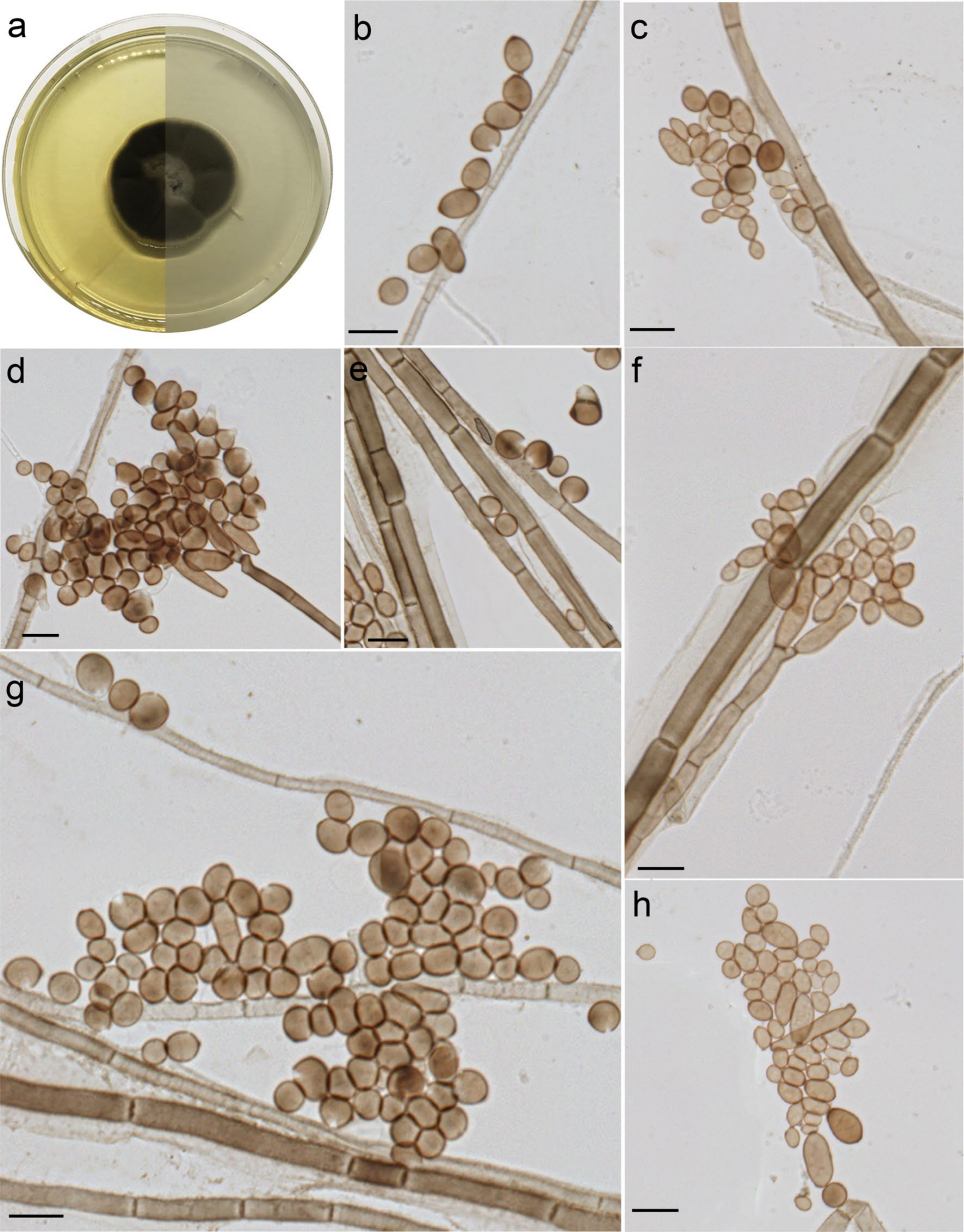
*Exophiala sacchari* microscopic morphology (UPCB 96825). **A** Colony on SGA; **B–E, G–H** Oval to lemon-shaped conidia; **F** Conidiophore. Bars = 10 μm

***Exophiala sacchari*** Costa, de Hoog, Gomes & Vicente, **sp. nov.** (Fig. 7).

MycoBank no.: MB839695.

*Etymology:* The name refers to the source of the isolation of this species, the sugarcane plant.

*Diagnosis*: The species differs from its nearest neighbour *E*. *pisciphila* sequences of ITS (98%), LSU (99%), and *BT2* (95%).

*Type:* **Brazil:** *São Paulo state*: Campinas city, greenhouse, 22.77584° W,47.09883 ^o^ S, isolated from rhizosphere of sugarcane (*Saccharum officinarum*, 5 Apr. 2018, *F.F. Costa* (UPCB 96825 – holotype [dried culture]; CMRP3436 ex-type culture). Sequences in GenBank: ITS (MZ132100.1), LSU (MW881154.1), *BT2* (ON455203), *TEF1* (OQ348494).

*Description: Macromorphology:* Colonies on SGA medium after 2 weeks incubation at 28 ºC moderately expanding and restricted, appearing velvety, smooth, convex with greyish tinge on the centre (the diameter reaches about 1 cm) and light brown tinge at the margin becoming black with time (1 mm thick). The reverse is olive black to black, with margin light brown, no pigment exuded on the agar. *Micromorphology:* Hyphae pale brown to brown, 2.4–3.9 μm wide, septate every 14–35 μm. Conidiogenous cells flask-shaped, mostly in loose clusters or branched systems. Conidia 2.0–14.1 × 2.6–6.6 μm, ellipsoidal, cylindrical, or lemon-shaped, 7.1 × 4.3 μm on average (Fig. 7).

*Note:* *Exophiala sacchari* is related to *E*. *pisciphila* in our phylogenetic analyses (Figs. 2 and 3). The genus *Exophiala* is polyphyletic with a wide distribution over the tree, and occupy different habitats including e.g. plants, seawater, and coconut shells (de Hoog et al. 2011; Nascimento et al. 2017). While *E*. *pisciphila* has been isolated as a pathogen of cold-blooded animals (de Hoog et al. 2011), *E*. *sacchari* originates from the sugarcane plant. In addition, *E*. *sacchari* has larger conidia without septation. Based on a BLASTn search in GenBank, the highest ITS similarity is with *E*. *pisciphila* [strain CBS 537.73, GenBank NR_121269.1; identities = 437/445 (98%), 0 gaps (0%)].

## PHYSIOLOGY

**Fig. 8:**
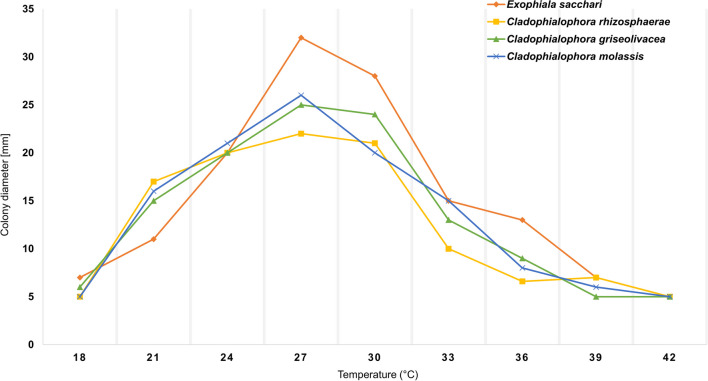
Temperature relations of *Cladophialophora* and *Exophiala* species after 3 wk incubation at temperatures ranging from 18 to 42 °C

The above environmental *Cladophialophora* isolates were phylogenetically remote from clinically relevant members of the genus. The strains showed optimal development at 27 °C, while growth was observed in the entire range between 19 and 37 °C. The isolates of *Exophiala* also showed optimal development at 27 °C, with growth between 21 and 37 °C. The maximum growth temperature of all strains analyzed was found to be 37 °C and no growth was observed at 40 °C (Fig. 8).

Considering the ecology of species evaluated, it was observed that *Cladophialophora bantiana* CMRP 3443 showed the ability to assimilate the following carbon sources: D-glucose, sucrose, maltose, soluble starch, and glycerol, but did not assimilate D-ribose, D- and L-arabinose, and meso-erythritol, and showed weak growth in remaining carbon sources (Table 4) after 14 days of incubation. Moderate halophily of CMRP 3443 was shown by growth at MgCl_2_ concentrations of 5% and 10% and weak growth with 5% NaCl. The strain *C. bantiana* (CMRP 3443) was able to assimilate all tested sugars, with optimal growth in the presence of sucrose, lactose, and soluble starch. Nitrogen assimilation abilities were evident by growth mainly in the presence of NaNO_3,_ KNO_3_ and L-lysine.

**Table 4:** Physiological tests of *Cladophialophora bantiana* CMRP 3443 with different carbon and nitrogen sources as well as concerning osmotolerance

Carbon sources:	Carbon sources:	Nitrogen sources:	Compounds:
Assimilation	Fermentation	Assimilation	Tolerance
D-glucose: +	Glucose: -	NaNO_3_: +	5.0% MgCl_2_: w/ +
D-ribose: −	Maltose: -	KNO_3_: w/ +	10.0% MgCl_2_: w/ +
L-arabinose: −	Sucrose: -	L-lysine: +	5.0% NaCl: w/ +
D-arabinose: −	Melibiose: -		10.0% NaCl: -
L-rhamnose: w/ +	Lactose: -		
Sucrose: +	Soluble starch: -		
Maltose: +			
Melibiose: w/ +			
Lactose: +			
Soluble starch: +			
Glycerol: +			
Meso-erythritol: -			
D-Mannitol: w/ +			
Ethanol: w/ +			

+  = Growth, w = Weak growth, − = No growth

*Cladophialophora bantiana* CMRP 3443 showed considerable tolerance of high sugar concentrations (Table 5). Tolerance of CMRP 3443 to increasing concentrations (30% and 60%) of sucrose and 30% of glucose was indicated by weak growth after 7 days compared to the control. The growth limit was around 30% of sugar; at higher concentrations, expansion growth was neglectable. Soluble starch did not affect growth in all concentrations evaluated. Biomass production in the presence of soluble starch was larger at all concentrations compared to glucose and sucrose.

**Table 5:** *Cladophialophora bantiana* CMRP 3443 tolerance of various sugars under increased concentration of glucose, sucrose, and soluble starch

	Day 2	Day 7	Day 14
Glucose 5%	+	+ +	+ +
Glucose 10%	w/ +	+ +	+ +
Glucose 30%	–	+	+
Glucose 60%	–	w/−	w/−
Sucrose 5%	+	+ +	+ +
Sucrose 10%	w	+ +	+ +
Sucrose 30%	–	w/-	w/-
Sucrose 60%	–	w/-	w/-
Soluble starch 5%	+	+	+ +
Soluble starch 10%	+	+	+ +
Soluble starch 30%	w/ +	+ +	+ +
Soluble starch 60%	w/ +	+ +	+ +

+  = Growth, w = Weak growth, − = No growth

## DISCUSSION

The black yeasts and their relatives comprise numerous agents of human and (mostly cold-blooded) animal infection (Hoog et al. 2011; Queiroz-Telles et al. 2017). In the environment, the species seem to occur in specific micro-habitats, and because of a low competitive ability towards other microorganisms, isolation of members of *Chaetothyriales* requires selective methods (Vicente et al. 2014). Oligotrophism is an important characteristic that enables them to survive at low density on adverse, low-nutrient substrates where common saprobes are absent (Vicente et al. 2001, 2008; Satow et al. 2008). Usually, agents of disease in *Chaetothyriales* are morphologically indistinguishable from their environmental relatives (Vicente et al. 2014), and thus reliable molecular and genomics tools are necessary for accurate identification of species (Vicente et al. 2017; Moreno et al. 2018).

In this study, we investigated the occurrence of black yeasts in sugarcane samples through in silico and in vitro methods. The culture-independent method applied was based on previous work of Souza et al. (2016), demonstrating the presence of *Chaetothyriales* in metagenome data from sugarcane. Using barcode and padlock probes proposed by Costa et al. (2020), it was possible to detect the presence of the genera *Cladophialophora*, *Cyphellophora*, *Exophiala*, *Knufia*, *Phialophora*, *Rhinocladiella,* and *Veronaea* in the sugarcane environment. The oil flotation isolation method after Vicente et al. (2014) was used to confirm the metagenomic identification of high numbers of black yeast-like fungi that were detected after *in-silico* analysis. The method was successfully applied in earlier environmental studies to recover etiologic agents of chromoblastomycosis and phaeohyphomycosis (Vicente et al. 2012, 2014; Salgado et al. 2004; Marques et al. 2006; Lima et al. 2020). In the present study, the method proved to be efficient for isolation of several potential agents of disease. *Rhinocladiella similis*, recently described as an agent of chromoblastomycosis (Heidrich et al. 2017) was isolated as an endophyte from sugarcane stalk, root, and leaf. In addition, *Exophiala spinifera* (Kapatia et al. 2019) was isolated from the root (endo- and exophytic) and *E. lecanii-corni* (Lee et al. 2016) from the sugarcane root. Moreover, *Exophiala cancerae* was found in sugarcane root, a species known as causal agent of Lethargic crab disease (LCD) in Brazil (de Hoog et al. 2011), confirming earlier environmental metagenomic data (Costa et al. 2020). *Cyphellophora oxyspora* (syn. *Phialophora oxyspora*)*,* described as an agent of mycetoma of a horse (Lopez et al. 2007), was recovered from sugarcane rhizosphere.

Surprisingly, among the species identified was the neurotropic fungus *Cladophialophora bantiana*, a pathogen almost entirely restricted to human brain (Badali et al. 2008), affecting immunocompromised as well as immunocompetent hosts (Kantarcioglu et al. 2017). The species causes primary brain infection (Horré and Hoog 1999), i.e., is acquired through inhalation but first symptoms are of neurological nature. Its environmental niche has remained enigmatic (Badali et al. 2008). Very few studies mentioned environmental occurrence, i.e., in bark and sawdust (Dixon et al. 1987), scrapings of a brick wall (Espinel-Ingroff et al*.* 1982), and in hot tub water (Jurjevic et al. 2013). These studies relied on morphological identification, while our study is the first with proof of occurrence based on molecular sequence data. The species is able to grow at 42 ºC, which is a beneficial growth temperature for clinical strains (Ganavalli & Raghavendra 2011). Sugarcane provides osmotic conditions. A high degree of osmotolerance was noted in our *C. bantiana* isolates, with sugar tolerance above 30%. Salt tolerance was not remarkable. The sugar tolerance of this species may be an important parameter in finding the source and route of infection of patients with this severe brain disorder. Thus far the disease has rarely been observed in Brazil, neither has sugarcane been associated with cases of chromoblastomycosis or phaeohyphomycosis (Sangwan et al. 2013; Bobba 2014; Rasamoelina et al. 2020). However, black yeast infections are not reportable diseases; cases of chromoblastomycosis and phaeohyphomycosis are probably treated by local health systems or may remain undiagnosed due to precarious conditions in the sugarcane fields (Alves 2006).

Sugarcane obviously is an overlooked substrate for *Chaetothyriales*. Isolation and in silico analysis showed a wide variety of fungi in the sugarcane habitat, including the known infectious species *Cladophialophora bantiana*, *Exophiala cancerae*, *E. spinifera,* and *Rhinocladiella similis.* Among the isolates recovered were also four species that we describe here as new to science. One of these belongs to the salmonis-clade (Figs. 2, 3), a cluster of waterborne species (de Hoog et al. 2011). In addition, three new *Cladophialophora* species were introduced, located along several *Cladophialophora* clades that contain primarily environmental fungi. In* Herpotrichiellaceae*, morphological features are highly variable within species, and are polyphyletic within the order *Chaetothyriales* (Quan et al. 2020). For this reason, description of phenotypes is significant to understand the ecology of the fungus at hand, but provides no reliable diagnostic features (Quan et al. 2023). Although the new species are morphologically hardly distinguishable from other species of the genera *Cladophialophora* and *Exophiala*, the multilocus analysis revealed a robust phylogenetic distance. This defines a better criterion to separate species in *Herpotrichiellaceae*. Furthermore, black yeasts have not previously been explored in sugarcane by selective isolation, which appears a major ecological feature of the new species.

Optimal temperature of growth for these species was 27 °C, with a mesophilic growth in the range 19–37 °C (Fig. 8). Despite the prevalence of infectious diseases by *Chaetothyriales* in Brazil, among which is chromoblastomycosis, only few of the potential etiologic agents have been recovered from the environment (Vicente et al. 2001, 2008, 2014). Marques et al. (2006) and Nascimento et al. (2017) were the first to observe high diversity and density of black yeasts on rotting Babassu coconut shells.

Many species remain undiscovered until their preferred habitat is found (Vicente et al. 2008). The genus *Cladophialophora* is characterized by melanized hyphae and absence of differentiated conidiophores with conidia produced sympodially in long, coherent chains. This character appeared polyphyletic within the *Chaetothyriales*. Many such species cause human infections, but others are purely environmental. Hence, distinction of opportunists and strict saprobes, as well as elucidation of environmental niches of the infectious species is mandatory to understand the ecology and epidemiology, and potential publish health risks of these fungi (de Hoog et al. 2011).

In this work, the *in-silico* analysis of sugarcane samples revealed a favorable environment for black yeasts, which was confirmed by selective isolation. The in silico re-analyses performed after introduction of the new species confirmed the presence of the novel taxa in the dataset. *Cyphellophora,* found as an abundant genus in the in silico analysis represented by *C. laciniata*, *C. suttonii,* and *C. vermispora,* which were not recovered in vitro. The same was observed for *Exophiala* spp*.* represented by 13 sequences in silico (Additional file 3: Table S3), while only *E. spinifera* and *E. cancerea* were isolated. Thus, studies based on molecular tools combined with in vitro isolation methods are fundamental for understanding the ecology of taxonomic groups that have not yet been fully elucidated.

Related species in the bantiana-clade can vary significantly in their ecological preferences and ability to cause infection in humans and animals (Vicente et al. 2014). Other abilities of these species concern the assimilation of a wide diversity of carbon sources (Tintelnot et al. 1995; de Hoog et al. 1995, 2004). Among the 14 carbon sources tested, the environmental lineage of *C*. *bantiana* did not assimilate D-ribose, L-arabinose, D-arabinose and meso-erythritol. Physiological profiles are similar to those of other species in the bantiana-clade, i.e., in *Fonsecaea pedrosoi* and *F*. *monophora*. Differences were noted with *C*. *bantiana* (CBS 173.52, CBS 364.80, CBS 328.65, CBS 564.82, and CBS 678.69) (de Hoog et al. 1995, 2004) in assimilation of ribose, L- and D-arabinose, and erythritol.

Judging from literature, the clinical strain of *C. bantiana* CBS 173.52 does not tolerate 10% MgCl_2_ and NaCl (de Hoog et al. 1995), while CMRP 3443 from the environment tolerated 5% and 10% MgCl_2_ and 5% NaCl, but not 10% NaCl. Halotolerance may be an important factor for the ubiquity of these strains in the phyllosphere of plants. The role of tolerance of high osmotic pressure in *C. bantiana* is thus far unexplained, but in general, extremotolerance is often a prerequisite for opportunism (Gostinčar et al. 2018).

## CONCLUSIONS

The results obtained in this study show that in silico methods such as metagenomics allied with barcodes directed the in vitro isolation to efficiently discover opportunistic black yeasts. With this combination of techniques, it was possible to establish the environmental niche of *C. bantiana,* as well as of other opportunistic species such as *Cyphellophora oxyspora, Exophiala cancerae*, *E. lecanii-corni*, *E. spinifera,* and *Rhinocladiella similis* in sugarcane. Four novel endophytic, saprobic black yeasts are introduced as new species in the family *Herpotrichiellaceae*. Future work might benefit from the use of metagenomics and barcodes, contributing to the elucidation of niches and to bioprospecting black yeasts in other substrates.

### Supplementary Information

**Additional file 1. Supplementary Figure F1.** Phylogeny of isolates from black yeast species.

**Additional file 2. Supplementary Table S1.** Molecular markers described in the literature of black yeasts

**Additional file 3. Supplementary Table S2.** Herpotrichiellaceae family reference strains used.

**Additional file 4. Supplementary Table S3.** Sequence quantities found in the in silico identification of sugarcane.

## Data Availability

All sequence data generated for this study (Table 3) can be accessed via. GenBank: https://www.ncbi.nlm.nih.gov/genbank/ and alignments are available at TreeBase (http:// www.treebase.org).

## Abbreviations

ATM: Atmosphere
BI: Bayesian inference
BT2: β-Tubulin
CBS: Fungal biodiversity centre
CIA: Acidic chloroform isoamyl alcohol solution
CMRP: The Microbiological Collections of Paraná NetworkCMRP/Taxonline
CTAB: Cetyl-trimethyl-ammonium bromide
dNTPs: Deoxynucleotide triphosphate
GPS: Global positioning system
ITS: Internal transcribed spacer
LCD: Lethargic crab disease
LSU: Large subunit of the nuclear ribosomal DNA
MAFFT: Multiple alignment using fast Fourier transform
MEA: Malt extract agar
MEGA: Molecular evolutionary genetics analysis
MgCl_2_: Magnesium chloride
ML: Maximum likelihood
NaCl: Sodium chloride
PCR: Polymerase chain reaction
PI: Parsimony informative
RPM: Revolutions per minute
SAP: Shrimp alkaline phosphatase
SGA: Sabouraud glucose agar
TEF1: Translation elongation factor 1-α
UPCB: Department of Botany Herbarium at the Federal University of Paraná

## References

[CR1] Alves F (2006). Por que morrem os cortadores de cana?. Saúde Soc.

[CR2] Arcobello JT, Revankar SG (2020). Phaeohyphomycosis. Semin Respir Crit Care Med.

[CR3] Badali H, Gueidan C, Najafzadeh MJ, Bonifaz A, Gerrits van den Ende AHG, de Hoog GS (2008). Biodiversity of the genus *Cladophialophora*. Stud Mycol.

[CR4] Bobba S (2014). Case study: chromoblastomycosis. J Trop Dis.

[CR5] Canilha L, Chandel AK, Suzane SMT, Antunes FAF, Luiz CFW, Das Graças AFM, Da Silva SS (2012). Bioconversion of sugarcane biomass into ethanol: an overview about composition, pretreatment methods, detoxification of hydrolysates, enzymatic saccharification, and ethanol fermentation. J Biomed Biotechnol.

[CR6] Carbone I, Kohn LM (1999). A method for designing primer sets for speciation studies in filamentous ascomycetes. Mycologia.

[CR7] Cheavegatti-Gianotto A, de Abreu HMC, Arruda P, Bespalhok FJC, Burnquist WL, Creste S, di Ciero L, Ferro JA, de Oliveira FAV, de Sousa FT, Grossi-de-Sá MF, Guzzo EC, Hoffmann HP, de Andrade LMG, Macedo N, Matsuoka S, de Castro RF, Romano E, da Silva WJ, de Castro SFM, César UE (2011). Sugarcane (*Saccharum X officinarum*): a reference study for the regulation of genetically modified cultivars in Brazil. Trop Plant Biol.

[CR8] Costa FF, da Silva NM, Voidaleski MF, Weiss VA, Moreno LF, Schneider GX, Najafzadeh MJ, Sun J, Gomes RR, Raittz RT, Castro MAA, de Muniz GBI, de Hoog GS, Vicente VA (2020). Environmental prospecting of black yeast-like agents of human disease using culture-independent methodology. Sci Rep.

[CR9] Cuadros-Orellana S, Leite LR, Smith A, Medeiros JD, Badotti F, Fonseca PLC, Vaz ABM, Oliveira G, Goes-Neto A (2013). Assessment of fungal diversity in the environment using metagenomics:a decade in review. Fung Genom Biol.

[CR10] De Arruda MR, Giller KE, Slingerland M (2017). Where is sugarcane cropping expanding in the Brazilian cerrado, and why? A case study. An Acad Bras Cienc.

[CR11] De Azevedo CMPS, Gomes RR, Vicente VA, Santos DWCL, Marques SG, Nascimento MMF, Andrade CEW, Silva RR, Queiroz-Telles F, De Hoog GS (2015). *Fonsecaea pugnacius*, a novel agent of disseminated chromoblastomycosis. J Clin Microbiol.

[CR12] Deng S, de Hoog GS, Pan W, Chen M, Gerrits van den Ende AHG, Yang L, Sun J, Najafzadeh MJ, Liao W, Li R (2014). Three isothermal amplification techniques for rapid Identification of *Cladophialophora carrionii*, an agent of human Chromoblastomycosis. J Clin Microbiol.

[CR13] de Hoog GS, Attili-Angelis D, Vicente VA, Gerrits van den Ende AHG, Queiroz-Telles F (2004). Molecular ecology and pathogenic potential of *Fonsecaea* species. Med Mycol.

[CR14] de Hoog GS, Guarro J, Gené J, Ahmed SA, Al-Hatmi AMS, Figueras MJ, Vitale RG (2000). Atlas of clinical fungi.

[CR15] de Hoog GS, Guého E, Masclaux F, Gerrits van den Ende AHG, Kwon-Chung KJ, McGinnis MR (1995). Nutritional physiology and taxonomy of human-pathogenic *Cladosporium-Xylohypha* species. J Med Vet Mycol.

[CR16] de Hoog GS, Vicente VA, Najafzadeh MJ, Harrak MJ, Badali H, Seyedmousavi S (2011). Waterborne *Exophiala* species causing disease in cold-blooded animals. Persoonia.

[CR17] de Lima BJF, S, Voidaleski MF, Gomes RR, Fornari G, Soares JMB, Bombassaro A, Schneider GX, Soley BS, de Azevedo CMPS, Menezes C, Moreno LF, Attili-Angelis D, Klisiowicz DR, de Hoog GS, Vicente VA, (2020). Selective isolation of agents of chromoblastomycosis from insect-associated environmental sources. Fungal Biol.

[CR18] Dixon DM, Merz WG, Elliott HL, Macleay S (1987). Experimental central nervous system phaeohyphomycosis following intranasal inoculation of *Xylohypha bantiana* in cortisone-treated mice. Mycopathologia.

[CR19] Duarte APM, Attili-Angelis D, Baron NC, Forti LC, Pagnocca FC (2014). Leaf-cutting ants: an unexpected microenvironment holding human opportunistic black fungi. Antonie Van Leeuwenhoek.

[CR20] Espinal-Ingroff A, Kerkering TM, Shadomy HJ (1982). Isolation of dematiaceous pathogenic fungi from a feed and seed warehouse. J Clin Microbiol.

[CR21] Feng P, Klaassen CHW, Meis JF, Najafzadeh MJ, Gerrits van den Ende AHG, Xi L, de Hoog GS (2013). Identification and typing of isolates of *Cyphellophora* and relatives by use of amplified fragment length polymorphism and rolling circle amplification. J Clin Microbiol.

[CR22] Ganavalli SA, Raghavendra DK (2012). *Cladophialophora bantiana*, the neurotropic fungus - a mini review. J Clin Diagnostic Res.

[CR23] Glass NL, Donaldson G (1995). Development of primer sets designed for use with PCR to amplify conserved genes from filamentous ascomycetes. Appl Environ Microbiol.

[CR24] Gomes RR, Vicente VA, Azevedo CMPS, Salgado CG, da Silva MB, Queiroz-Telles F, Marques SG, Santos DWCL, de Andrade TS, Takagi EH, Cruz KS, Fornari G, Hahn RC, Scroferneker ML, Caligine RB, Ramirez-Castrillon M, de Araújo DP, Heidrich D, Colombo AL, de Hoog GS (2016). Molecular epidemiology of agents of human chromoblastomycosis in Brazil with the description of two novel species. PLoS Negl Trop Dis.

[CR25] Gostinčar C, Zajc J, Lenassi M, Plemenitaš A, de Hoog GS, Al-Hatmi AMS, Gunde-Cimerman N (2018). Fungi between extremotolerance and opportunistic pathogenicity on humans. Fungal Divers.

[CR26] Hall TA (1999). BioEdit: a user-friendly biological sequence alignment editor and analysis program for Windows 95/98. Nucl Acids Symp.

[CR27] Hamzehei H, Yazdanparast SA, Mohammad DM, Khodavaisy S, Golehkheyli M, Ansari S, de Hoog GS, Badali H (2013). Use of rolling circle amplification to rapidly identify species of *Cladophialophora* potentially causing human infection. Mycopathologia.

[CR28] Heidrich D, González GM, Pagani DM, Ramírez-Castrillón M, Scroferneker ML (2017). Chromoblastomycosis caused by *Rhinocladiella similis*: case report. Med Mycol Case Rep.

[CR29] Heinrichs G, de Hoog GS, Haase G (2012). Barcode identifiers as a practical tool for reliable species assignment of medically important black yeast species. J Clin Microbiol.

[CR30] Horré R, de Hoog GS (1999). Primary cerebral infections by melanized fungi: a review. Stud Mycol.

[CR31] Isola D, Selbmann L, de Hoog GS, Fenice M, Onofri S, Prenafeta-Boldú FX, Zucconi L (2013). Isolation and screening of black fungi as degraders of volatile aromatic hydrocarbons. Mycopathologia.

[CR32] Iwatsu T, Miyaji M, Okamoto S (1981). Isolation of *Phialophora verrucosa* and *Fonsecaea pedrosoi* from nature in Japan. Mycopathologia.

[CR33] Jurjevic Z, Li C, Dobranic J (2013) First finding of *Cladophialophora bantiana* from the indoor environment in the United States. In: Abstracts of the American industrial hygeine conference & exposition, Montréal, Canada

[CR34] Kantarcioglu AS, Guarro J, de Hoog GS, Apaydin H, Kiraz N (2017). An updated comprehensive systematic review of *Cladophialophora bantiana* and analysis of epidemiology, clinical characteristics, and outcome of cerebral cases. Med Mycol.

[CR35] Kapatia G, Pandey T, Kakkar N, Kaur H, Verma R (2019). Facial phaeohyphomycosis in an immunocompetent individual: a rare presentation of a rare fungus. Am J Dermatopathol.

[CR36] Katoh K, Rozewicki J, Yamada KD (2018). MAFFT online service: multiple sequence alignment, interactive sequence choice and visualization. Brief Bioinform.

[CR37] Kumar P, Barrett DM, Delwiche MJ, Stroeve P (2009). Methods for pretreatment of lignocellulosic biomass for efficient hydrolysis and biofuel production. Ind Eng Chem Res.

[CR38] Kumar S, Stecher G, Tamura K (2016). MEGA7: molecular evolutionary genetics analysis version 7.0 for bigger datasets. Mol Biol Evol.

[CR39] Lee KC, Kim MJ, Chae SY, Lee HS, Jang YH, Lee SJ, Kim DW, Lee WJ (2016). A case of phaeohyphomycosis caused by *Exophiala lecanii-corni*. Ann Dermatol.

[CR40] Lima MAC, Garcia RDO, Martins GS, Mansur E (2001). Morfogênese *in vitro* e susceptibilidade de calos de variedades nacionais de cana-de-açúcar (*Saccharum officinarum* L.) a agentes seletivos utilizados em sistemas de transformação genética. Rev Bras Botânica.

[CR41] Lima J dos S (2008) Diversidade genética e rnadf de isolados de *Colletotrichum* spp. endofíticos da planta medicinal *Schinus terebinthifolius* Raddi (Aroeira) 84. Thesis, Federal University of Paraná

[CR42] Lopez MJ, Robinson SO, Cooley AJ, Prichard MA, Mcginnis MR (2007). As the cause of mycetoma in a horse. J Amer Vet Med Assoc.

[CR43] Marques SG, Silva CDMP, Saldanha PC, Rezende MA, Vicente VA, Queiroz-Telles F, Costa JML (2006). Isolation of *Fonsecaea pedrosoi* from the shell of the babassu coconut (*Orbignya phalerata* Martius) in the amazon region of Maranhão Brazil. J Med Mycol.

[CR44] Moreno LF, Feng P, Weiss VA, Vicente VA, Stielow JB, de Hoog GS (2017). Phylogenomic analyses reveal the diversity of laccase-coding genes in *Fonsecaea* genomes. PLoS ONE.

[CR45] Moreno LF, Vicente VA, de Hoog GS (2018). Black yeasts in the omics era: achievements and challenges. Med Mycol.

[CR46] Najafzadeh MJ, Dolatabadi S, Saradeghi KM, Naseri A, Feng P, de Hoog GS (2013). Detection and identification of opportunistic *Exophiala* species using the rolling circle amplification of ribosomal internal transcribed spacers. J Microbiol Methods.

[CR47] Najafzadeh MJ, Sun J, Vicente VA, de Hoog GS (2011). Rapid identification of fungal pathogens by rolling circle amplification using *Fonsecaea* as a model. Mycoses.

[CR48] Najafzadeh MJ, Vicente VA, Feng P, Naseri A, Sun J, Rezaei-Matehkolaei A, de Hoog GS (2018). Rapid identification of seven waterborne *Exophiala* species by RCA DNA padlock probes. Mycopathologia.

[CR49] Nascimento MMF, Vicente VA, Bittencourt JVM, Gelinski JML, Prenafeta-Boldú FX, Romero-Güiza M, Fornari G, Gomes RR, Santos GD, Gerrits van den Ende AHG, de Azevedo CDMPS, de Hoog GS (2017). Diversity of opportunistic black fungi on babassu coconut shells, a rich source of esters and hydrocarbons. Fungal Biol.

[CR50] O'Donnell K (1992). Ribosomal DNA internal transcribed spacers are highly divergent in the phytopathogenic ascomycete *Fusarium sambucinum* (*Gibberella pulicaris*). Curr Genet.

[CR51] Petrini O (1991) Fungal endophytes of tree leaves. Microb Ecol Leaves 179–180

[CR52] Quan Y, Deng S, Prenafeta-Boldủ FX, Mayer VE, Muggia L, Cometto A, Vicente VA, Silva NM, Grisolia ME, Song Y, Ahmed AS, Niu X, Lima BJFS, Feng P, Vitale RG, Teixeira M, Sudhadham M, de Azevedo CPS, Bocca A, Hasse G, Selbmann SD, Kang Y, de Hoog GS (2023). The origin of human pathogenicity and biological interactions in *Chaetothyriales*. Fungal Divers.

[CR53] Quan Y, Muggia L, Moreno LF, Wang M, Al-Hatmi AMS, da Silva NM, Shi D, Deng S, Ahmed S, Hyde KD, Vicente VA, Kang Y, Stielow JB, de Hoog GS (2020). A re-evaluation of the *Chaetothyriales* using criteria of comparative biology. Fungal Divers.

[CR54] Queiroz-Telles F, de Hoog GS, Santos DW, Salgado CG, Vicente VA, Bonifaz A, Roilides E, Xi L, Azevedo CM, da Silva MB, Pana ZD, Colombo AL, Walsh TJ (2017). Chromoblastomycosis. Clin Microbiol Rev.

[CR55] Rasamoelina T, Maubon D, Andrianarison M, Ranaivo I, Sendrasoa F, Rakotozandrindrainy N, Rakotomalala FA, Bailly S, Rakotonirina B, Andriantsimahavandy A, Rabenja FR, Andrianarivelo MR, Cornet M, Ramarozatovo LS (2020). Endemic chromoblastomycosis caused predominantly by *Fonsecaea nubica*, Madagascar. Emerg Infect Dis.

[CR56] Revankar SG, Baddley JW, Chen SCA, Kauffman CA, Slavin M, Vazquez JA, Seas C, Morris MI, Nguyen MH, Shoham S, Thompson GR, Alexander BD, Simkins J, Ostrosky-Zeichner L, Mullane K, Alangaden G, Andes DR, Cornely OA, Wahlers K, Lockhart SR, Pappas PG (2017). A mycoses study group international prospective study of phaeohyphomycosis: an analysis of 99 proven/probable cases. Open Forum Infect Dis.

[CR57] Salgado CG, Da Silva JP, Diniz JAP, Da Silva MB, Da Costa PF, Teixeira C, Salgado UI (2004). Isolation of *Fonsecaea pedrosoi* from thorns of *Mimosa pudica*, a probable natural source of chromoblastomycosis. Rev Inst Med Trop Sao Paulo.

[CR58] Sangwan J, Lathwal S, Juyal D, Sharma N (2013). *Fonsecaea pedrosoi*: A rare etiology in fungal keratitis. J Clin Diagn Res.

[CR59] Santos DWCL, Vicente VA, Weiss VA, de Hoog GS, Gomes RR, Batista EMM, Marques SG, Queiroz-Telles F, Colombo AL, Azevedo CMPES (2020). Chromoblastomycosis in an endemic area of Brazil: a clinical-epidemiological analysis and a worldwide haplotype network. J Fungi.

[CR60] Satow MM, Attili-Angelis D, de Hoog GS, Angelis DF, Vicente VA (2008). Selective factors involved in oil flotation isolation of black yeasts from the environment. Stud Mycol.

[CR61] Schneider GX, Gomes RR, Bombassaro A, Zamarchi K, Voidaleski MF, Costa FF, Leão ACR, Lima BJFS, Soley BS, Colombo IR, Cândido GZ, Najafzadeh MJ, Sun J, de Azevedo CMPS, Marques SG, de Hoog GS, Vicente VA (2019). New molecular markers distinguishing *Fonsecaea* agents of chromoblastomycosis. Mycopathologia.

[CR62] Song Y, Silva NM, Vicente VA, Quan Y, Teixeira M, Gong J, de Hoog GS, Li R (2021). Comparative genomics of opportunistic *Phialophora* species involved in divergent disease types. Mycoses.

[CR63] Souza RSC, Okura VK, Armanhi JSL, Jorrín B, Lozano N, Da Silva MJ, González-Guerrero M, De Araújo LM, Verza NC, Bagheri HC, Imperial J, Arruda P (2016). Unlocking the bacterial and fungal communities assemblages of sugarcane microbiome. Sci Rep.

[CR64] Teixeira MM, Moreno LF, Stielow BJ, Muszewska A, Hainaut M, Gonzaga L, Abouelleil A, Patané JSL, Priest M, Souza R, Young S, Ferreira KS, Zeng Q, da Cunha MML, Gladki A, Barker B, Vicente VA, de Souza EM, Almeida S, Henrissat B, Vasconcelos ATR, Deng S, Voglmayr H, Moussa TAA, Gorbushina A, Felipe MSS, Cuomo CA, de Hoog GS (2017). Exploring the genomic diversity of black yeasts and relatives (Chaetothyriales, *Ascomycota*). Stud Mycol.

[CR65] Tintelnot K, von Hunnius P, de Hoog GS, Polak-Wyss A, Guého E, Masclaux F (1995). Systemic mycosis caused by a new *Cladophialophora* species. Med Mycol.

[CR66] Vaezi A, Fakhim H, Abtahian Z, Khodavaisy S, Geramishoar M, Alizadeh A, Meis JF, Badali H (2018). Frequency and geographic distribution of CARD9 mutations in patients with severe fungal infections. Front Microbiol.

[CR67] Vicente VA, Attili-Angelis D, Pie MR, Queiroz-Telles F, Cruz LM, Najafzadeh MJ, de Hoog GS, Zhao J, Pizzirani-Kleiner A (2008). Environmental isolation of black yeast-like fungi involved in human infection. Stud Mycol.

[CR68] Vicente VA, Attili-Angelis D, Queiróz-Telles F, Pizzirani-Kleiner A (2001). Isolation of herpotrichiellaceous fungi from the environment. Braz J Microbiol.

[CR69] Vicente VA, Najafzadeh MJ, Sun J, Gomes RR, Robl D, Marques SG, Azevedo CMPS, De Hoog GS (2014). Environmental siblings of black agents of human chromoblastomycosis. Fungal Divers.

[CR70] Vicente VA, Orélis-Ribeiro R, Najafzadeh MJ, Sun J, Guerra RS, Miesch S, Ostrensky A, Meis JF, Klaassen CH, de Hoog GS, Boeger WA (2012). Black yeast-like fungi associated with lethargic crab disease (LCD) in the mangrove-land crab, *Ucides cordatus* (*Ocypodidae*). Vet Microbiol.

[CR71] Vicente VA, Weiss VA, Bombassaro A, Moreno LF, Costa FF, Raittz RT, Leão AC, Gomes RR, Bocca AL, Fornari G, Castro RJA, Sun J, Faoro H, Tadra-Sfeir MZ, Balsanelli BV, E, Almeida SR, dos Santos SS, Teixeira MM, Felipe MSS, Nascimento MMF, Pedrosa FO, Steffens MB, Attili-Angelis D, Najafzadeh MJ, Queiroz-Telles F, Souza EM, de Hoog GS, (2017). Comparative genomics of sibling species of *Fonsecaea* associated with human chromoblastomycosis. Front Microbiol.

[CR72] Vilgalys R, Hester M (1990). Rapid genetic identification and mapping of enzymatically amplified ribosomal DNA from several *Cryptococcus* species. J Bacteriol.

[CR73] Wang X, Cai W, Gerrits van den Ende AHG, Zhang J, Xie T, Xi L, Li X, Sun J, de Hoog GS (2018). Indoor wet cells as a habitat for melanized fungi, opportunistic pathogens on humans and other vertebrates. Sci Rep.

[CR74] White TJ, Bruns TD, Lee SB, Taylor JW, Innis MA, Gelfand DH, Sninsky JJ, White TJ (1990). Amplification and direct sequencing of fungal ribosomal RNA genes for phylogenetics. PCR protocols: a guide to methods and applications.

