# Supplementary material for: Sugarcane: an unexpected habitat for black yeasts in Chaetothyriales
Source: IMA Fungus. 2023 Oct 4;14:20. doi: 10.1186/s43008-023-00124-7 (PMC10552356; doi:10.1186/s43008-023-00124-7)
Supplement: Supplementary file 1 — Additional file 1. Supplementary Figure F1. Phylogeny of isolates from black yeast species. [file 43008_2023_124_MOESM1_ESM.docx]

**Sugarcane: an unexpected habitat for black yeasts in Chaetothyriales**

**Flávia de F. Costa^1^ • Rafael S. C. de Souza^2^ • Morgana F. Voidaleski^3^ • Renata R. Gomes^3^ • Guilherme F. Reis^1^ • Bruna J. F. de S. Lima^3^ • Giovanna Z. Candido^3^ • Marlon R. Geraldo^3^ • Jade M. B. Soares^4^ • Gabriela X. Schneider^3^ • Edvaldo da S. Trindade^5^ • Israel H. Bini^5^ • Leandro F. Moreno^3^ • Amanda Bombassaro^3^ • Flávio Queiroz-Telles^3,6^ • Roberto T. Raittz^7^ • Yu Quan^8^ • Paulo Arruda^2,9^ • Derlene A. de Angelis^10^ • Sybren de Hoog^3,8*^ • Vania A. Vicente^1, 3,*^**

^1^ Engineering Bioprocess and Biotechnology Post-Graduation Program, Department of Bioprocess Engineering and Biotechnology, Federal University of Paraná, Curitiba, Paraná, Brazil

^2^ Molecular Biology and Genetics Engineering Center, State University of Campinas (UNICAMP), Campinas, São Paulo, Brazil

^3^ Microbiology, Parasitology and Pathology Post-Graduation Program, Department of Basic Pathology, Federal University of Paraná, Curitiba, Paraná, Brazil

^4^ Biological Sciences Graduation, Federal University of Paraná, Curitiba, Paraná, Brazil

^5^ Department of Cell Biology, Federal University of Paraná, Curitiba, Brazil

^6^ Clinical Hospital of the Federal University of Paraná, Curitiba, Brazil

^7^ Laboratory of Bioinformatics, Professional and Technological Education Sector, Federal University of Paraná, Curitiba, Brazil

^8^ Center of Expertise in Mycology of Radboud, University Medical Center / Canisius Wilhelmina Hospital, Nijmegen, The Netherlands

^9^ Genetics and Evolution Department, Biology Institute, State University of Campinas (UNICAMP), Campinas, São Paulo, Brazil

^10^ Division of Microbial Resources (DRM/CPQBA), University of Campinas, Campinas, Brazil

**Supplementary Figure F1.** Phylogeny of isolates from black yeast species.


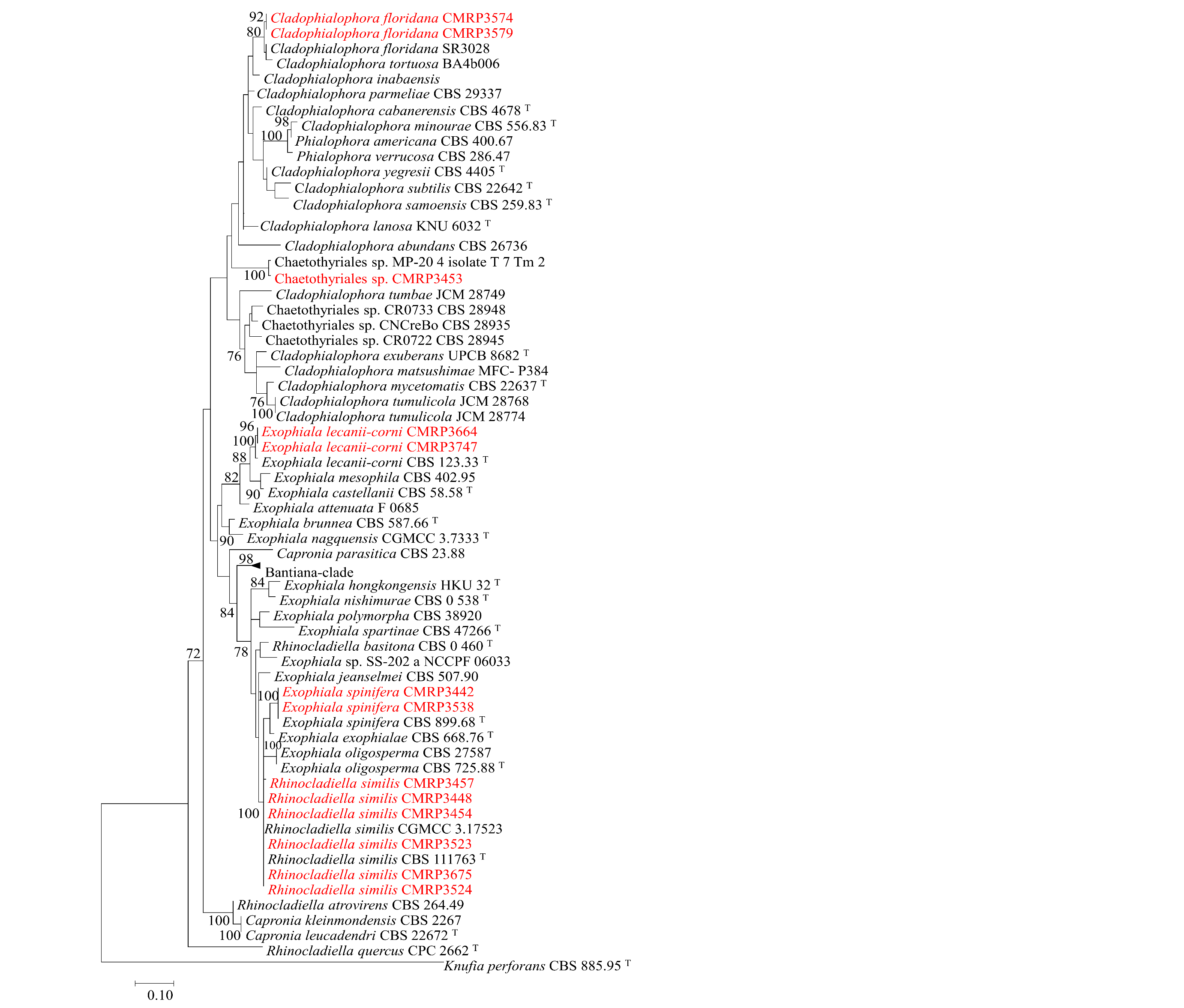


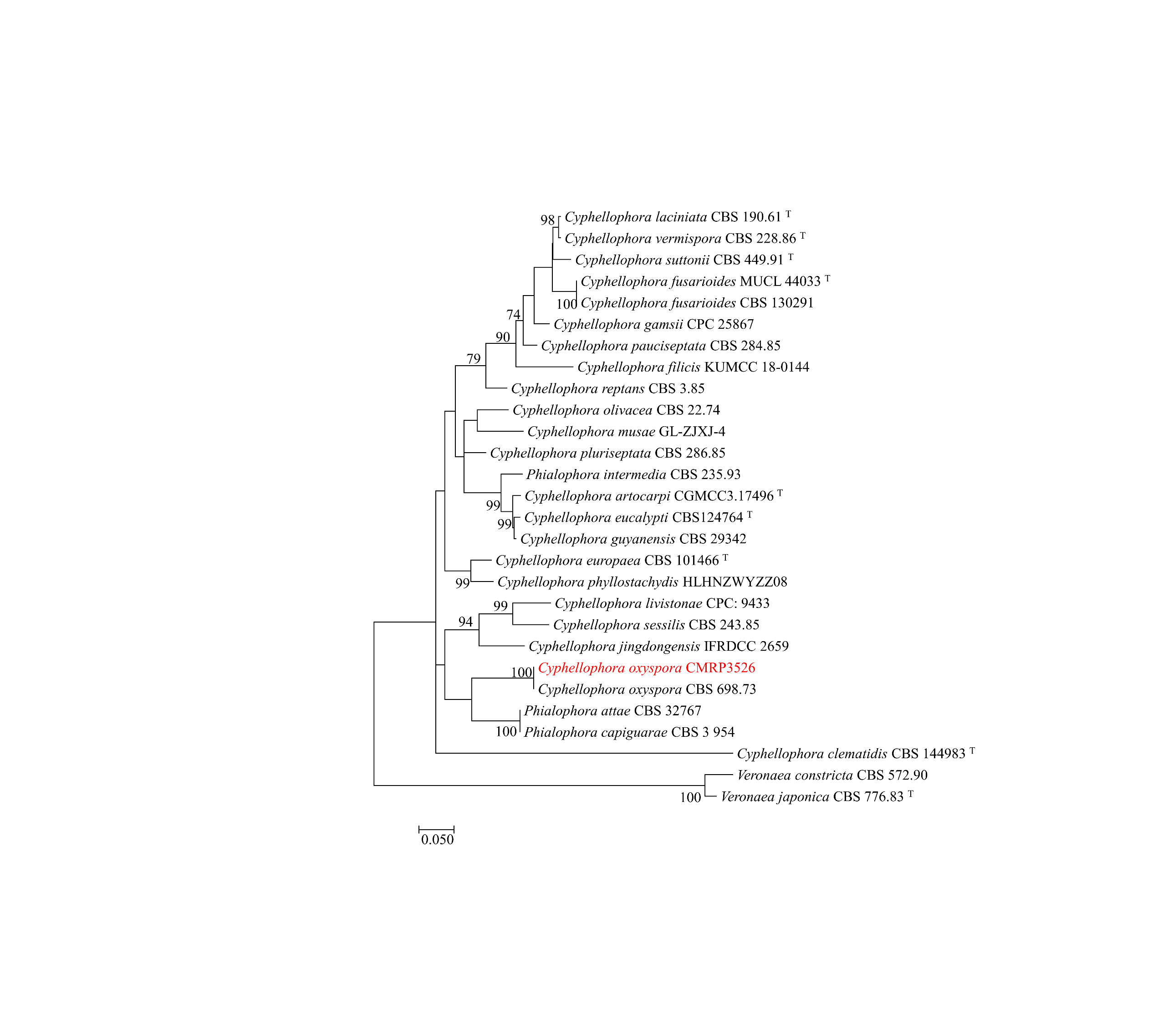


**Fig. S1** Phylogeny of a representative selection of species in Chaetothyriales order based on confidently aligned sequences. Constructed with Maximum likelihood implemented in MEGA 7. Bootstrap values > 70% from 1000 resampled datasets are shown with branches. T = Type strain.
