# Supplementary material for: Sugarcane: an unexpected habitat for black yeasts in Chaetothyriales
Source: IMA Fungus. 2023 Oct 4;14:20. doi: 10.1186/s43008-023-00124-7 (PMC10552356; doi:10.1186/s43008-023-00124-7)
Supplement: Supplementary file 2 — Additional file 2. Supplementary Table S1. Molecular markers described in the literature of black yeasts [file 43008_2023_124_MOESM2_ESM.docx]

**Sugarcane: an unexpected habitat for black yeasts in Chaetothyriales**

**Flávia de F. Costa^1^ • Rafael S. C. de Souza^2^ • Morgana F. Voidaleski^3^ • Renata R. Gomes^3^ • Guilherme F. Reis^1^ • Bruna J. F. de S. Lima^3^ • Giovanna Z. Candido^3^ • Marlon R. Geraldo^3^ • Jade M. B. Soares^4^ • Gabriela X. Schneider^3^ • Edvaldo da S. Trindade^5^ • Israel H. Bini^5^ • Leandro F. Moreno^3^ • Amanda Bombassaro^3^ • Flávio Queiroz-Telles^3,6^ • Roberto T. Raittz^7^ • Yu Quan^8^ • Paulo Arruda^2,9^ • Derlene A. de Angelis^10^ • Sybren de Hoog^3,8*^ • Vania A. Vicente^1, 3,*^**

^1^ Engineering Bioprocess and Biotechnology Post-Graduation Program, Department of Bioprocess Engineering and Biotechnology, Federal University of Paraná, Curitiba, Paraná, Brazil

^2^ Molecular Biology and Genetics Engineering Center, State University of Campinas (UNICAMP), Campinas, São Paulo, Brazil

^3^ Microbiology, Parasitology and Pathology Post-Graduation Program, Department of Basic Pathology, Federal University of Paraná, Curitiba, Paraná, Brazil

^4^ Biological Sciences Graduation, Federal University of Paraná, Curitiba, Paraná, Brazil

^5^ Department of Cell Biology, Federal University of Paraná, Curitiba, Brazil

^6^ Clinical Hospital of the Federal University of Paraná, Curitiba, Brazil

^7^ Laboratory of Bioinformatics, Professional and Technological Education Sector, Federal University of Paraná, Curitiba, Brazil

^8^ Center of Expertise in Mycology of Radboud, University Medical Center / Canisius Wilhelmina Hospital, Nijmegen, The Netherlands

^9^ Genetics and Evolution Department, Biology Institute, State University of Campinas (UNICAMP), Campinas, São Paulo, Brazil

^10^ Division of Microbial Resources (DRM/CPQBA), University of Campinas, Campinas, Brazil

**Supplementary Table S1.** Molecular markers described in the literature of black yeasts.

| Species | Strains | Source | Number pb | | Ref. |
| --- | --- | --- | --- | --- | --- |
|  |  |  | Barcodes | Probes padlock |  |
| *Cladophialophora arxii* | CBS 306.94^T^ | Human | 35 | 42 | ^1, 2^ |
|  | CBS 409.96 | Human | 37 | - | ^1^ |
| *Cladophialophora bantiana* | CBS 173.52 | Human | 34 | 41 | ^1, 2^ |
| *Cladophialophora chaetospira* | CBS 115468 | Plant | - | 39 | ^2^ |
| *Cladophialophora carrionii* | CBS 160.54^LT^ | Human | 29 | 42 /39 | ^1-3^ |
|  | CBS 163.54 | Human | 29 | - | ^1^ |
|  | CBS 260.83 | Human | 29 | - | ^1^ |
|  | FMC.248 | Human | 29 | - | ^1^ |
| *Cladophialophora devriesii* | CBS 147.84^T^ | Human | 25 | 42 | ^1, 2^ |
|  | IFM51369 | - | 25 | - | ^1^ |
| *Cladophialophora emmonsii* | CBS 979.96^T^ | Human | 33 | - | ^1^ |
|  | CBS 640.96 | Cat | 33 | - | ^1^ |
| *Cladophialophora immunda* | CBS 834.96^T^ | Human | 25 | 41 / 42 | ^1,2^ |
|  | CBS 102227 | Plant | 25 | - | ^1^ |
| *Cladophialophora minourae* | CBS 987.96 | Rotting wood | - | 42 | ^2^ |
| *Cladophialophora modesta* | CBS 985.96^T^ | Human | 26 | - | ^1^ |
| *Cladophialophora mycetomatis* | CBS 122637^T^ | Human | 28 | - | ^1^ |
|  | CBS 454.82 | Culture contaminant | 28 | - | ^1^ |
| *Cladophialophora psammophila* | CBS 110553^T^ | Gasoline-polluted soil | 34 | 41 | ^1, 2^ |
| *Cladophialophora samoensis* | CBS 259.83^T^ | Human | 31 | - | ^1^ |
| *Cladophialophora saturnica* | CBS 118724^T^ | Child | 27 | 42 | ^1,2^ |
| *Cladophialophora subtilis* | CBS 12264^T^ | Iced tea | 30 | - | ^1^ |
| *Cladophialophora yegresii* | CBS 114405^T^ | Plant | 30 | 42 | ^1,2^ |
| *Cyphellophora guyanensis* | CBS 124764T | Plant | - | 42 | ^8^ |
| *Cyphellophora laciniata* | CBS190.61^T^ | Human | 35 | 34 | ^1,8^ |
| *Cyphellophora pluriseptata* | CBS286.85^T^ | Human | 31 | 36 | ^1,8^ |
| *Cyphellophora suttonii* | CBS449.91^T^ | Dog ear | 35 | - | ^1^ |
| *Cyphellophora vermispora* | CBS228.86^T^ | Plant | 35 | 32 | ^1,8^ |
| *Exophiala alcalophila* | CBS 520.82^T^ | Soil | 31 | - | ^1^ |
| *Exophiala angulospora* | CBS 482.92^T^ | Water | 28 | 36 | ^1, 4^ |
|  | CBS 122264 | Hydrocarbon-polluted soil | 28 | - | ^1^ |
|  | CBS 109906 | Water | 28 | - | ^1^ |
| *Exophiala aquamarina* | CBS 119918^T^ | Seadragon, skin | 38 | 37 | ^1, 4^ |
| *Exophiala asiatica* | BMU00015^T^ | Human | 36 | - | ^1^ |
| *Exophiala attenuata* | CBS110026 | Human | 29 | - | ^1^ |
| *Exophiala bergeri* | CBS 353.52^T^ | Human | 31 | - | ^1^ |
| *Exophiala brunnea* | CBS 587.66^T^ | Plant | 38 | - | ^1^ |
| *Exophiala cancerae* | CBS 120532^T^ | Crab | 34 | - | ^1^ |
| *Exophiala castellanii* | CBS 158.58^T^ | Human | 28 | 37 | ^1,4^ |
| *Exophiala capensis* | CBS 128771^T^ | Plant | 31 | - | ^1^ |
| *Exophiala dermatitidis* | CBS 207.35^T^ | Human | 32 | 36 | ^1, 5^ |
|  | CBS 100338 | Humidifier | 32 | - | ^1^ |
|  | BMU00035 | Human | 32 | - | ^1^ |
| *Exophiala equina* | CBS 119.23^T^ | Horse skin | 38 | - | ^1^ |
|  | CBS 122263 | Human foot | 38 | - | ^1^ |
|  | CBS 122270 | Human foot | 38 | - | ^1^ |
| *Exophiala exophialae* | CBS 668.76^T^ | Straw in burrow | 32 | - | ^1^ |
| *Exophiala halophila* | CBS 121512^T^ | Human | 34 | - | ^1^ |
| *Exophiala heteromorpha* | CBS 232.33^T^ | Wood pulp | 29 | - | ^1^ |
| *Exophiala jeanselmei* | CBS 507.90^T^ | Human | 36 | 37 | ^1, 5^ |
|  | CBS 677.76 | Human | 37 | - | ^1^ |
| *Exophiala lacus* | CBS 117497^T^ | Water | 37 | - | ^1^ |
| *Exophiala lecanii-corni* | CBS 123.33^T^ | Human | 29 | - | ^1^ |
| *Exophiala mesophila* | CBS 402.95^T^ | Shower | 28 | - | ^1^ |
|  | CBS 121511 | Human | 28 | - | ^1^ |
| *Exophiala moniliae* | CBS 520.76^T^ | Plant | 26 | - | ^1^ |
| *Exophiala nishimurae* | CBS 101538^T^ | Bark | 32 | - | ^1^ |
| *Exophiala oligosperma* | CBS 725.88^T^ | Human | 38 | 37 | ^1, 5^ |
|  | UTHSC95-2041 | Human | 38 | - | ^1^ |
|  | UTHSC91###870 | Human | 39 | - | ^1^ |
|  | IFM41701 | Soil | 40 | - | ^1^ |
| *Exophiala opportunistica* | CBS109811 | Water | 39 | 36 | ^1,4^ |
| *Exophiala pisciphila* | CBS 537.73^T^ | Fish | 39 | 37 | ^1, 4^ |
| *Exophiala salmonis* | CBS 157.67^T^ | Fish | 37 | - | ^1^ |
| *Exophiala sideris* | CBS 121818^T^ | Plant | 29 | - | ^1^ |
| *Exophiala spinifera* | CBS 899.68^T^ | Human | 32 | 34 | ^1,5^ |
| *Exophiala siphonis* | UTHSC88-471 | Human | 27 | - | ^1^ |
| *Exophiala xenobiotica* | CBS 118157^T^ | Oil sludge | 30 | 37 | ^1, 5^ |
|  | CBS 119306 | Human | 30 | - | ^1^ |
|  | CBS 117665 | Human | 30 | - | ^1^ |
|  | CBS 117676 | Human | 33 | - | ^1^ |
|  | CBS 117641 | Human | 32 | - | ^1^ |
| *Fonsecaea erecta* | dH20513 | Plant | 33 | - | ^1^ |
|  | dH20502 | Plant | 33 | - | ^1^ |
| *Fonsecaea minima* | dH20511 | Plant | 33 | - | ^1^ |
| *Fonsecaea monophora* | CBS 269.37^T^ | Human | 36 | 28 | ^1, 6^ |
|  | CBS 121732 | Human | 36 | - | ^1^ |
|  | IFM4889 | Human | 36 | - | ^1^ |
|  | IFM54446 | Human | 36 | - | ^1^ |
| *Fonsecaea multimorphosa* | CBS 980.96^T^ | Cat | 27 | - | ^1^ |
| *Fonsecaea nubica* | CBS 269.64^T^ | Human | 36 | 36 | ^1, 6^ |
|  | CBS 121733 | Human | 36 | - | ^1^ |
|  | CBS 557.76 | Unknown | 36 | - | ^1^ |
| *Fonsecaea pedrosoi* | CBS 271.37^T^ | Human | 36 | 36 | ^1, 6^ |
|  | CBS 122741 | Human | 37 | - | ^1^ |
| *Fonsecaea pugnacius* | CBS 139214 | Human | - | 31 | ^7^ |
| *Knufia epidermidis* | CBS120353^T^ | Human | 30 | - | ^1^ |
| *Phialophora americana* | CBS 840.69 | Decaying timber | 30 | - | ^1^ |
| *Phialophora europaea* | CBS129.96^T^ | Human | 18 | 34 | ^1,8^ |
| *Phialophora oxyspora* | CBS 698.73^T^ | Plant | - | 37 | ^8^ |
| *Phialophora reptans* | CBS113.85^T^ | Food | 30 | 37 | ^1,8^ |
| *Phialophora verrucosa* | CBS 273.37 | Human | 30 | - | ^1^ |
|  | IMTSP.800 | Soil | 30 | - | ^1^ |
|  | CBS 286.47 | Plant | 30 | - | ^1^ |
|  | CBS 839.68 | Kiwifruit elephantiasis | 30 | - | ^1^ |
| *Rhinocladiella anceps* | CBS 181.65 | Soil | 30 | - | ^1^ |
|  | CBS 157.54 | Plant | 32 | - | ^1^ |
| *Rhinocladiella aquaspersa* | CBS 313.73^T^ | Human | 27 | - | ^1^ |
| *Rhinocladiella atrovirens* | CBS 264.49 | Honey | 28 | - | ^1^ |
| *Rhinocladiella basitona* | CBS 101460^T^ | Human | 31 | - | ^1^ |
| *Rhinocladiella mackenziei* | CBS 650.93^T^ | Human | 37 | - | ^1^ |
|  | CBS 367.92 | Human | 35 | - | ^1^ |
|  | CBS 102590 | Human | 35 | - | ^1^ |
| *Rhinocladiella similis* | CBS 111763^T^ | Human | 32 | - | ^1^ |
|  | dH13054 | Water | 32 | - | ^1^ |
| *Veronaea botryosa* | CBS 254.57^T^ | Sansa olive slag | 40 | 35 | ^1, 4^ |
|  | CBS 350.65 | Dung of goat | 41 | - | ^1^ |
| *Veronaea compacta* | CBS 268.75^T^ | Unknown | 38 | - | ^1^ |
| *Veronaea japonica* | CBS 776.83^T^ | Dead bamboo culm | 38 | - | ^1^ |

## References

1. Heinrichs, G., De Hoog, G. S. & Haase, G. Barcode identifiers as a practical tool for reliable species assignment of medically important black yeast species. *J. Clin. Microbiol.* **50**, 3023–3030, 10.1128/JCM.00574-12 (2012).

2. Hamzehei, H. *et al.* Use of rolling circle amplification to rapidly identify species of *Cladophialophora* potentially causing human infection. *Mycopathologia.* **175**, 431–438, 10.1007/s11046-013-9630-7 (2013).

3. Deng, S. *et al.* Three isothermal amplification techniques for rapid identification of *Cladophialophora carrionii*, an agent of human chromoblastomycosis. *J. Clin. Microbiol.* **52**, 3531–3535, 10.1128/JCM.01033-14 (2014).

4. Najafzadeh, M. J. *et al.* Rapid identification of seven waterborne Exophiala species by RCA DNA padlock probes. *Mycopathologia.* **183**, 669–677, 10.1007/s11046-018-0256-7 (2018).

5. Najafzadeh, M. J. *et al.* Detection and identification of opportunistic *Exophiala* species using the rolling circle amplification of ribosomal internal transcribed spacers. *J. Microbiol. Methods.* **94**, 338–342, 10.1016/j.mimet.2013.06.026 (2013).

6. Najafzadeh, M. J., Sun, J., Vicente, V. A. & de Hoog, G. S. Rapid identification of fungal pathogens by rolling circle amplification using *Fonsecaea* as a model. *Mycoses.* **54**, 577–582, 10.1111/j.1439-0507.2010.01995.x (2011).

7. Schneider, G. X. *et al.* New molecular markers distinguishing *Fonsecaea* agents of chromoblastomycosis. *Mycopathologia.* **184**, 493–504, 10.1007/s11046-019-00359-2 (2019).

8. Feng, P., Klaassen, C. H., Meis, J. F., Najafzadeh, M. J., Gerrits Van Den Ende, A. H., Xi, L., & de Hoog, G. S. (2013). Identification and typing of isolates of Cyphellophora and relatives by use of amplified fragment length polymorphism and rolling circle amplification. Journal of clinical microbiology, 51(3), 931–937. https://doi.org/10.1128/JCM.02898-12
