# Supplementary material for: Sugarcane: an unexpected habitat for black yeasts in Chaetothyriales
Source: IMA Fungus. 2023 Oct 4;14:20. doi: 10.1186/s43008-023-00124-7 (PMC10552356; doi:10.1186/s43008-023-00124-7)
Supplement: Supplementary file 3 — Additional file 3. Supplementary Table S2. Herpotrichiellaceae family reference strains used. [file 43008_2023_124_MOESM3_ESM.docx]

**Sugarcane: an unexpected habitat for black yeasts in Chaetothyriales**

**Flávia de F. Costa^1^ • Rafael S. C. de Souza^2^ • Morgana F. Voidaleski^3^ • Renata R. Gomes^3^ • Guilherme F. Reis^1^ • Bruna J. F. de S. Lima^3^ • Giovanna Z. Candido^3^ • Marlon R. Geraldo^3^ • Jade M. B. Soares^4^ • Gabriela X. Schneider^3^ • Edvaldo da S. Trindade^5^ • Israel H. Bini^5^ • Leandro F. Moreno^3^ • Amanda Bombassaro^3^ • Flávio Queiroz-Telles^3,6^ • Roberto T. Raittz^7^ • Yu Quan^8^ • Paulo Arruda^2,9^ • Derlene A. de Angelis^10^ • Sybren de Hoog^3,8*^ • Vania A. Vicente^1, 3,*^**

^1^ Engineering Bioprocess and Biotechnology Post-Graduation Program, Department of Bioprocess Engineering and Biotechnology, Federal University of Paraná, Curitiba, Paraná, Brazil

^2^ Molecular Biology and Genetics Engineering Center, State University of Campinas (UNICAMP), Campinas, São Paulo, Brazil

^3^ Microbiology, Parasitology and Pathology Post-Graduation Program, Department of Basic Pathology, Federal University of Paraná, Curitiba, Paraná, Brazil

^4^ Biological Sciences Graduation, Federal University of Paraná, Curitiba, Paraná, Brazil

^5^ Department of Cell Biology, Federal University of Paraná, Curitiba, Brazil

^6^ Clinical Hospital of the Federal University of Paraná, Curitiba, Brazil

^7^ Laboratory of Bioinformatics, Professional and Technological Education Sector, Federal University of Paraná, Curitiba, Brazil

^8^ Center of Expertise in Mycology of Radboud, University Medical Center / Canisius Wilhelmina Hospital, Nijmegen, The Netherlands

^9^ Genetics and Evolution Department, Biology Institute, State University of Campinas (UNICAMP), Campinas, São Paulo, Brazil

^10^ Division of Microbial Resources (DRM/CPQBA), University of Campinas, Campinas, Brazil

**Supplementary Table S2**. Herpotrichiellaceae family reference strains used.

| **Species** | **Access number** | **Status** | **GenBank ID** | | | |
| --- | --- | --- | --- | --- | --- | --- |
|  |  |  | **LSU** | **ITS** | **Β-tubulin** | **TEF1** |
| *Aculeata aquatica* | MFLUCC 11-0529 | Type strain | MG922571 | MG922575 | - | - |
| *Capronia camelliae yunnanensis* | CGMCC 3 19061 | Type strain | NG066425 | NR164589 | - | - |
| *Capronia coronata* | ATCC 56201 | Type strain | AF050242 | NR154745 | - | - |
| *Capronia dactylotricha* | CBS 604.96 | Type strain | KX712343 | AF050243 | - | - |
| *Capronia fungicola* | CBS 614 96 | Type strain | NG058761 | KY484990 | - | - |
| *Capronia kleinmondensis* | CBS 122671 | Type strain | EU552107 | MH863226 | - | - |
| *Capronia leucadendri* | CBS 122672 | Type strain | MH874754 | NR_156212 | - | - |
| *Capronia mansonii* | CBS 101.67 | Type strain | AY004338 | AF050247 | - | - |
| *Capronia moravica* | CBS 603.96 |  | KX712344 | - | - | - |
| *Capronia munkii* | AFTOL ID 656 |  | EF413604 | MH862601 | - | - |
| *Capronia parasitica* | CBS 123.88 |  | FJ358225 | AF050252 | - | - |
| *Capronia pilosella* | AFTOL657 |  | DQ823099 | DQ826737 | - | - |
| Chaetothyriales sp. | TRN531 |  | FJ358267 | - | - | - |
| Chaetothyriales sp. | TRN486 |  | FJ358261 | - | - | - |
| *Cladophialophora abundans* | CBS 126736 | Type strain | KC812100 | KC776592 | - | - |
| *Cladophialophora aquatica* | MC03A | Type strain | MT860433 | MT864355 | - | - |
| *Cladophialophora arxii* | CBS 306.94 | Type strain | NG058959 | EU103987 | EU137192 | - |
| *Cladophialophora australiensis* | CBS 112793 | Type strain | EU035402 | EU137331 | - | - |
| *Cladophialophora bantiana* | CBS 173.52 | Type strain | KF155189 | EU103989 | - | - |
| *Cladophialophora bantiana* | CBS 100429 |  | MH877849 | KF155212 | - | - |
| *Cladophialophora boppii* | CBS 126.86 | Type strain | NG058762 | MH861932 | - | - |
| *Cladophialophora bromeliacearum* | URM 8085 | Type strain | MW794274 | - | - | - |
| *Cladophialophora bromeliacearum* | FCCUFG 04 |  | MW794275 | - | - | - |
| *Cladophialophora chaetospira* | CBS 491.70 | Type strain | EU035405 | EU035403 | KF928578 | - |
| *Cladophialophora cabanerensis* | CBS 146718 | Type strain | NG073760 | NR169978 | - | - |
| *Cladophialophora carrionii* | CBS 160.54 | Type strain | NG 055741 | MH857278 | XM08730518 | XM08724425 |
| *Cladophialophora carrionii* | CBS 163.54 |  | KF928515 | MH857279 | KF928579 | EU137244 |
| *Cladophialophora chinesis* | KUMCC 21-0209 | Type strain | MZ420202 | MZ420207 | MZ419853 | MZ419851 |
| *Cladophialophora devriesii* | CBS 147.84 | Type strain | KC809989 | EU103985 | - | - |
| *Cladophialophora emmonsii* | CBS 640.96 | Type strain | KC809995 | EU103995 | - | - |
| *Cladophialophora exuberans* | CMRP1227 | Type strain | KY570931 | NG060431 | KY689826 | - |
| *Cladophialophora exuberans* | CMRP1219 |  | KY570930 | KY680430 | KY689827 | - |
| *Cladophialophora floridana* | SR3028 | Type strain | AB986343 | AB986343 | - | - |
| *Cladophialophora floridana* | SR1004 |  | AB986344 | AB986344 | - | - |
| *Cladophialophora griseolivacea* | CMRP3446 | Type strain | MW861546 | MZ048747 | ON553224 | OQ348498 |
| *Cladophialophora griseolivacea* | CMRP3441 |  | MW861545 | MZ029088 | ON553225 | OQ348499 |
| *Cladophialophora immunda* | CBS 834.96 | Type strain | KC809990 | MH862619 | EU137257 |  |
| *Cladophialophora matsushimae* | MFC-1P384 | Type strain | FN400758 | FN549916 | - | - |
| *Cladophialophora minourae* | CBS 556.83 | Type strain | NG058763 | AY251087 | - | - |
| *Cladophialophora minourae* | BMU 05999 |  | KJ930159 | KJ701016 | - | - |
| *Cladophialophora molassis* | CMRP3450 | Type strain | MW865735 | MZ132103 | ON455204 | OQ348500 |
| *Cladophialophora molassis* | CMRP3461 |  | MW865734 | MZ126811 | ON455205 | OQ348501 |
| *Cladophialophora multiseptata* | CBS 136675 | Type strain | NG064270 | NR132894 | - | - |
| *Cladophialophora mycetomatis* | CBS 122637 | Type strain | NG058960 | FJ385276 | - | - |
| *Cladophialophora mycetomatis* | CBS 454.82 |  | LC192077 | EU137293 | - |  |
| *Cladophialophora nyingchiensis* | CGMCC317330 | Type strain | MG197824 | MG012699 | MG012747 | MG012706 |
| *Cladophialophora nyingchiensis* | CGMCC317514 |  | - | MG01270 | MG012749 | MG012708 |
| *Cladophialophora nyingchiensis* | CGMCC317329 |  | - | MG012700 | MG012748 | MG012707 |
| *Cladophialophora parmeliae* | CBS 129337 | Type strain | JQ342182 | JQ342180 | - | - |
| *Cladophialophora psammophila* | CBS 110553 | Type strain | NG058955 | AY857517 | XM07745883 | - |
| *Cladophialophora potulentorum* | CBS 114772 |  | EU035410 | EU035410 | - | - |
| *Cladophialophora potulentorum* | CBS 112222 |  | EU035409 | - | - | - |
| *Cladophialophora pseudocarrionii* | CBS 138591 | Type strain | KU705844 | - | - | - |
| *Cladophialophora recurvata* | FMR 16667 | Type strain | NG075226 | NR172270 | - |  |
| *Cladophialophora rhizosphaerae* | CMRP3553 | Type strain | MW856019 | MZ006214 | ON553222 | OQ348496 |
| *Cladophialophora rhizosphaerae* | CMRP3556 |  | MW715827 | MZ008436 | ON553223 | OQ348497 |
| *Cladophialophora samoensis* | CBS 259.83 | Type strain | NG058854 | MH861581 | EU137174 | - |
| *Cladophialophora saturnica* | CBS 102230 |  | KC809993 | - | - | - |
| *Cladophialophora subtilis* | CBS 122642 | Type strain | NG058961 | FJ385273 | - | - |
| *Cladophialophora tengchongensis* | CGMCC 3.15201 | Type strain | MG197827 | MG012702 | MG012750 | - |
| *Cladophialophora tortuosa* | BA4b006 | Type strain | AB986424 | AB986424 | - | - |
| *Cladophialophora tumbae* | JCM 28749 | Type strain | LC192072 | LC192125 | - | - |
| *Cladophialophora tumbae* | JCM 28753 |  | LC192073 | - | - | - |
| *Cladophialophora tumulicola* | JCM 28766 | Type strain | LC192063 | LC192127 | - | - |
| *Cladophialophora tumulicola* | JCM 28774 |  | LC192065 | - | - | - |
| *Cladophialophora yegresii* | CBS 114405 | Type strain | NG058855 | EU137322 | EU137209 | XM007760340 |
| *Cyphellophora artocarpi* | CGMCC3.17496 | Type strain | - | KP010367 | - | - |
| *Cyphellophora clematidis* | CBS 144983 | Type strain | - | MK442577 | - | - |
| *Cyphellophora eucalypti* | CBS124764 | Type strain | - | GQ303274 | - | - |
| *Cyphellophora europaea* | CBS 101466 | Type strain | - | JQ766443 | - | - |
| *Cyphellophora filicis* | KUMCC 18-0144 |  | - | MK404056 | - | - |
| *Cyphellophora fusarioides* | MUCL 44033 | Type strain | - | NR132879 | - | - |
| *Cyphellophora fusarioides* | CBS 130291 |  | - | JQ766439 | - | - |
| *Cyphellophora gamsii* | CPC 25867 | Type strain | - | KX228255 | - | - |
| *Cyphellophora laciniata* | CBS 190.61 | Type strain | - | JQ766423 | - | - |
| *Cyphellophora vermispora* | CBS 228.86 | Type strain | - | KC455244 | - | - |
| *Cyphellophora suttonii* | CBS 449.91 | Type strain | - | JQ766459 | - | - |
| *Cyphellophora pauciseptata* | CBS 284.85 | Type strain | - | JQ766466 | - | - |
| *Cyphellophora oxyspora* | CBS 698.73 | Type strain | - | MH860790 | - | - |
| *Cyphellophora oxyspora* | CMRP3526 |  | - | MT331614 | - | - |
| *Cyphellophora sessilis* | CBS 243.85 |  | - | MH861875 | - | - |
| *Exophiala abietophila* | CBS 145038 | Type strain | NG066323 | NR163357 | - | - |
| *Exophiala alcalophila* | CBS 520.82 | Type strain | NG059189 | MH861524 | JN112423 | JN128771 |
| *Exophiala angulospora* | CBS 482.92 | Type strain | KF155190 | JF747046 | JN112426 | JN128780 |
| *Exophiala attenuata* | F10685 |  | KT013094 | KT013095.1 | - | - |
| *Exophiala aquamarina* | FMR 3998 |  | KU705846 | KX712347 | JN112434 | - |
| *Exophiala aquamarina* | R-3685 |  | KX712347 |  |  | - |
| *Exophiala bergeri* | CBS 353.52 | Type strain | NG059199 | MH857080 | EF551497 | - |
| *Exophiala bonariae* | CCFEE 5792 | Type strain | KR781083 | JX681046 | - | - |
| *Exophiala brunnea* | CBS 587.66 | Type strain | KX712342 | MH858890 | JN112442 | JN128783 |
| *Exophiala campbellii* | NCPF 2274 |  | LT594760 | LT594703 | - | - |
| *Exophiala cancerae* | CBS 110371 |  | KF155179 | - | - | - |
| *Exophiala cancerae* | CBS 120420 | Type strain | - | HQ659023 | JN112444 | JN128800 |
| *Exophiala cancerae* | CBS 117491 |  | - | KF928439 | JN112446 | JN128799 |
| *Exophiala cancerae* | CBS 115142 |  | - | JF747067 | - | - |
| *Exophiala capensis* | CBS 128771 | Type strain | NG059207 | NR121493 | - | - |
| *Exophiala castellanii* | CBS 158.58 | Type strain | KF928522 | MH857734 | KF928586 | JN128766 |
| *Exophiala cinerea* | CGMCC 3.18778 | Type strain | MG197820 | - | - | - |
| *Exophiala clavispora* | CGMCC3.17512 |  | MG197830 | NG074892 | KP347932 | - |
| *Exophiala clavispora* | CGMCC3.17513 |  | MG197830 | - | - | - |
| *Exophiala dermatitidis* | CBS 207.35 | Type strain | NG059225 | MH855649 | KF928572 | - |
| *Exophiala embothrii* | CBS146560 |  | MW045823 | MW045819 | MW055978 | - |
| *Exophiala embothrii* | CBS 146558 | Type strain | MW045821 | - | - | - |
| *Exophiala equina* | CBS 116009 | Type strain | KF928497 | KF928433 | KF928561 |  |
| *Exophiala equina* | CBS 128222 |  | KF928496 | - | - | - |
| *Exophiala ellipsoidea* | CGMCC3.17348 | Type strain | KP347956 | - | - | - |
| *Exophiala eucalypti* | CPC 27630 | Type strain | KY173502 | KY173502 | - | - |
| *Exophiala eucalypticola* | CBS 143412 | Type strain | NG063955 | MH107891 | MH108039 |  |
| *Exophiala exophialae* | CBS 668.76 | Type strain | KX822326 | - | - | - |
| *Exophiala frigidotolerans* | CBS 146539 | Type strain | LR699567 | LR699566 | LR699568 | - |
| *Exophiala heteromorpha* | CBS 232.33 | Type strain | NG0639751 | NR111184 | - | - |
| *Exophiala hongkongensis* | HKU 32 | Type strain | NG059264 | JN625231 | JN625236 | - |
| *Exophiala italica* | MFLUCC 160245 | Type strain | KY496723 | KY496744 | - | - |
| *Exophiala jeanselmei* | CBS 507.90 | Type strain | KJ930161 | AY156963 | EF551503 | - |
| *Exophiala lecanii-corni* | CBS 123 33 | Type strain | FJ358243 | MH855383 | - |  |
| *Exophiala lacus* | CBS 117497_ | Type strain | - | JF747110 | - | JN128776 |
| *Exophiala lacus* | FMR 3995 |  | KU705847 | KU705830 | - | - |
| *Exophiala lavatrina* | NCPF 7893 |  | LT594755 | LT594758 | - | - |
| *Exophiala lignicola* | CBS 144622 | Type strain | NG066324 | NR163358 | - | - |
| *Exophiala mali* | CBS 146791 | Type strain | MW175381 | MW175341 | - | - |
| *Exophiala mesophila* | CBS 402.95 |  | KX712349 | MH862536 | JN112476 | - |
| *Exophiala moniliae* | CBS 520.76 | Type strain | KJ930162 | KF881967 | - | - |
| *Exophiala nagquensis* | CGMCC:3.17284 |  | MG197838 | KP347960 | KP347924 | - |
| *Exophiala nidicola* | FMR 3889 | Type strain | MG701056 | MG701056 | - | - |
| *Exophiala nishimurae* | CBS 101538 | Type strain | KX712351 | - | - | - |
| *Exophiala nigra* | CBS 535.94 | Type strain | NG059253 | KY115191 | - | - |
| *Exophiala oligosperma* | CBS 728.88 | Type strain | FJ358245 | MH864631 | KF928550 | - |
| *Exophiala opportunistica* | CBS 122268 |  | KF928500 | KF928436 | KF928564 | - |
| *Exophiala palmae* | CMRP1196 | Type strain | NG064428 | NR158414 | KY689829 | - |
| *Exophiala phaeomuriformis* | MY458/2011 |  | JN165755 | - | - | - |
| *Exophiala pisciphila* | CBS 537.73 | Type strain | NR121269 | DQ826739 | JN112493 | - |
| *Exophiala pisciphila* | AFTOL-ID 669 |  | DQ823101 | DQ823101 | - | - |
| *Exophiala polymorpha* | CBS 138920 | Type strain | KP070764 | KP070763 | - | - |
| *Exophiala prostantherae* | CPC 38251 | Type strain | MW175384 | MW175344 | - | - |
| *Exophiala psychrophila* | CBS 191.87 | Type strain | MH873750 | MH873750 | JN112497 | JN128798 |
| *Exophiala psychrophila* | CBS 256.92 |  | - | JF747136 | JN112498 | - |
| *Exophiala pseudooligosperma* | YMF 16741 |  | MW616559 | - | - | - |
| *Exophiala quercina* | CPC33408 | Type strain | NG073874 | MT223892 | - | - |
| *Exophiala radicis* | CBS 140402 | Type strain | NG069319 | NG069319 | KT723463 | - |
| *Exophiala radicis* | P1910 |  | KT723446 | KT099204 | KT723462 | - |
| *Exophiala sacchari* | CMRP3436 | Type strain | MW881154 | MZ132100 | ON455203 | OQ348494 |
| *Exophiala sacchari* | CMRP3434 |  | MW881155 | MZ130934 | ON454893 | OQ348495 |
| *Exophiala salmonis* | CBS 157.67 | Type strain | MH870616 | JF747137 | JN112499 | - |
| *Exophiala salmonis* | CBS 120274 |  | KF928498 | KF928434 | KF928562 | - |
| *Exophiala sideris* | CBS 127096 |  | MH875856 | - | - | - |
| *Exophiala spinifera* | CBS 899.68 | Type strain | MH870977 | MH010942 | KF928553 | - |
| *Exophiala tremulae* | UAMH10998 | Type strain | F951155 | MH865222 | KT894148 | - |
| *Exophiala xenobiotica* | CBS 128104 |  | MH876272 | MH864829 | - | - |
| *Exophiala yunnanensis* | YMF1.06739 |  | MZ779229 | - | - | - |
| *Exophiala yuxiensis* | YMF1.07354 | Type strain | OL863154 | - | - | - |
| *Fonsecaea brasiliensis* | BMU 07620 |  | KJ930163 | - | - | - |
| *Fonsecaea erecta* | CBS 125763 | Type strain | KF155186 | KC886414 | KF155221 | - |
| *Fonsecaea minima* | CBS 125757 | Type strain | KF928520 | MH863743 | KF155222 | - |
| *Fonsecaea monophora* | CBS 269.37 | Type strain | KF155184 | AY857511 | EU938547 | - |
| *Fonsecaea multimorphosa* | CBS 980.96 | Type strain | NG057983 | NR111612 | HQ681121 | - |
| *Fonsecaea pedrosoi* | CBS 271.37 | Type strain | KJ930166 | AB114127 | EU938559 | - |
| *Fonsecaea pugnacius* | CBS 139214 | Type strain | NG058177 | NR155089 | KR706547 | - |
| *Knufia epidermidis* | CBS 120353 |  | NG042475 | NR111330 | - | - |
| *Phialophora americana* | CBS 400.67 |  | MH870708 | MH859007 | EU514708 | - |
| *Phialophora americana* | dH24528 |  | - | MT344076 | MT353670 | - |
| *Phialophora americana* | dH24533 |  | - | MT344071 | MT353665 | - |
| *Phialophora expanda* | CBS 140298 | Type strain | NG064295 | - | - | - |
| *Phialophora macrospora* | MUCL 9760 | Type strain | AF050281 | - | - | - |
| *Phialophora verrucosa* | CBS 140325 | Type strain | - | NR146242 | KF971761 | - |
| *Phialophora verrucosa* | CBS 140299 |  | - | KJ701008 | KM658115 | - |
| *Phialophora verrucosa* | CBS 140324 |  | - | KJ700942 | KM658087 | - |
| *Phialophora verrucosa* | CBS 286.47 |  | KF928519 | KF928455 | KF928583 | - |
| *Rhinocladiella anceps* | AFTOL ID 659 |  | DQ823102 | DQ826740 | - | - |
| *Rhinocladiella atrovirens* | CBS 264.49 | Type strain | EU041869 | MH856518 | - | - |
| *Rhinocladiella basitona* | CBS 101460 | Type strain | NG057783 | - | - | - |
| *Rhinocladiella coryli* | CPC 26654 | Type strain | KX306793 | KX306768 | - | - |
| *Rhinocladiella fasciculata* | CBS 132.86 | Type strain | NG057784 | NR145356 | - | - |
| *Rhinocladiella mackenziei* | CBS 650.93 | Type strain | AF050288 | - | - | - |
| *Rhinocladiella phaeophora* | CBS 496.78 | Type strain | NG057785 | EU041811 | GU079661 | - |
| *Rhinocladiella quercus* | CPC 26621 | Type strain | NG059698 | KX306769 | - | - |
| *Rhinocladiella similis* | PW3041 |  | LC158635 | LC158611 | - | - |
| *Rhinocladiella tropicalis* | RA776 | Type strain | KX356663 | KU854928 | - | - |
| *Veronaea aquatica* | JAUCC2549 | Type strain | MW046893 | - | - | - |
| *Veronaea compacta* | CBS 268.75 | Type strain | NG057790 | NR077176 | - | - |
| *Veronaea japonica* | CBS 776.83 | Type strain | NG057789 | MH861692 | - | - |
| *Veronaea botryosa* | CBS 254.57 | Type strain | MH869255 | MH857711 | JN112505 | - |
| *Veronaea botryosa* | UTHSCSA DI15439 |  | - | MH167396 | - | - |
| *Veronaea constricta* | CBS 572 90 |  | MH873920 | MH862237 | - | - |
