# Supplementary material for: Sugarcane: an unexpected habitat for black yeasts in Chaetothyriales
Source: IMA Fungus. 2023 Oct 4;14:20. doi: 10.1186/s43008-023-00124-7 (PMC10552356; doi:10.1186/s43008-023-00124-7)
Supplement: Supplementary file 4 — Additional file 4. Supplementary Table S3. Sequence quantities found in the in silico identification of sugarcane. [file 43008_2023_124_MOESM4_ESM.docx]

**Sugarcane: an unexpected habitat for black yeasts in Chaetothyriales**

**Flávia de F. Costa^1^ • Rafael S. C. de Souza^2^ • Morgana F. Voidaleski^3^ • Renata R. Gomes^3^ • Guilherme F. Reis^1^ • Bruna J. F. de S. Lima^3^ • Giovanna Z. Candido^3^ • Marlon R. Geraldo^3^ • Jade M. B. Soares^4^ • Gabriela X. Schneider^3^ • Edvaldo da S. Trindade^5^ • Israel H. Bini^5^ • Leandro F. Moreno^3^ • Amanda Bombassaro^3^ • Flávio Queiroz-Telles^3,6^ • Roberto T. Raittz^7^ • Yu Quan^8^ • Paulo Arruda^2,9^ • Derlene A. de Angelis^10^ • Sybren de Hoog^3,8*^ • Vania A. Vicente^1, 3,*^**

^1^ Engineering Bioprocess and Biotechnology Post-Graduation Program, Department of Bioprocess Engineering and Biotechnology, Federal University of Paraná, Curitiba, Paraná, Brazil

^2^ Molecular Biology and Genetics Engineering Center, State University of Campinas (UNICAMP), Campinas, São Paulo, Brazil

^3^ Microbiology, Parasitology and Pathology Post-Graduation Program, Department of Basic Pathology, Federal University of Paraná, Curitiba, Paraná, Brazil

^4^ Biological Sciences Graduation, Federal University of Paraná, Curitiba, Paraná, Brazil

^5^ Department of Cell Biology, Federal University of Paraná, Curitiba, Brazil

^6^ Clinical Hospital of the Federal University of Paraná, Curitiba, Brazil

^7^ Laboratory of Bioinformatics, Professional and Technological Education Sector, Federal University of Paraná, Curitiba, Brazil

^8^ Center of Expertise in Mycology of Radboud, University Medical Center / Canisius Wilhelmina Hospital, Nijmegen, The Netherlands

^9^ Genetics and Evolution Department, Biology Institute, State University of Campinas (UNICAMP), Campinas, São Paulo, Brazil

^10^ Division of Microbial Resources (DRM/CPQBA), University of Campinas, Campinas, Brazil

**Supplementary Table S3.** Sequence quantities found in the *in silico* identification of sugarcane.

| Species | Compartments of sugar cane | | | | | | | | | |  | |
| --- | --- | --- | --- | --- | --- | --- | --- | --- | --- | --- | --- | --- |
|  | Rhizosphere | Leaf | | Bottom stalk | | Medium Stalk | | Upper stalk | |  | |  |
|  |  | Endo | Exo | Endo | Exo | Endo | Exo | Endo | Exo | Total | |  |
| *Cladophialophora bantiana* | 13 | 0 | 0 | 43 | 14 | 0 | 1 | 0 | 0 | 71 | |  |
| *Cyphellophora laciniata* | 13 | 17 | 12 | 0 | 60 | 0 | 83 | 0 | 36 | 221 | |  |
| *Cyphellophora suttonii* | 2 | 0 | 9 | 0 | 74 | 24 | 7 | 0 | 11 | 127 | |  |
| *Cyphellophora vermispora* | 13 | 10 | 10 | 0 | 58 | 0 | 80 | 0 | 32 | 203 | |  |
| *Exophiala alcalophila* | 0 | 0 | 0 | 0 | 0 | 0 | 0 | 0 | 2 | 2 | |  |
| *Exophiala bergeri* | 14 | 0 | 24 | 0 | 38 | 0 | 86 | 0 | 2 | 164 | |  |
| *Exophiala brunnea* | 0 | 0 | 0 | 0 | 0 | 0 | 0 | 0 | 1 | 1 | |  |
| *Exophiala cancerae* | 165 | 0 | 0 | 0 | 13 | 0 | 62 | 0 | 0 | 240 | |  |
| *Exophiala dermatitidis* | 0 | 0 | 1 | 0 | 0 | 0 | 2 | 0 | 0 | 3 | |  |
| *Exophiala exophialae* | 0 | 0 | 0 | 58 | 118 | 108 | 208 | 0 | 74 | 566 | |  |
| *Exophiala heteromorpha* | 0 | 0 | 0 | 0 | 0 | 0 | 9 | 0 | 0 | 9 | |  |
| *Exophiala jeanselmei* | 0 | 0 | 0 | 0 | 1 | 0 | 0 | 0 | 0 | 1 | |  |
| *Exophiala oligosperma* | 0 | 0 | 0 | 0 | 9 | 0 | 77 | 0 | 0 | 87 | |  |
| *Exophiala pisciphila* | 353 | 0 | 0 | 10 | 57 | 46 | 12 | 101 | 0 | 579 | |  |
| *Exophiala sideris* | 0 | 0 | 0 | 0 | 3 | 0 | 2 | 0 | 0 | 5 | |  |
| *Exophiala spinifera* | 318 | 26 | 136 | 304 | 350 | 202 | 398 | 305 | 502 | 2541 | |  |
| *Exophiala xenobiotica* | 99 | 0 | 7 | 0 | 73 | 0 | 196 | 0 | 2 | 377 | |  |
| *Knufia epidermidis* | 0 | 3 | 2 | 0 | 0 | 0 | 3 | 0 | 0 | 5 | |  |
| *Phialophora verrucosa* | 2 | 0 | 0 | 0 | 0 | 0 | 0 | 0 | 0 | 2 | |  |
| *Rhinocaldiella similis* | 0 | 4 | 0 | 52 | 131 | 23 | 101 | 4 | 256 | 571 | |  |
| *Veronaea botryosa* | 22 | 0 | 0 | 0 | 20 | 0 | 14 | 0 | 3 | 59 | |  |
| Total | 1,014 | 57 | 201 | 467 | 1,019 | 403 | 1,341 | 410 | 921 | 5,834 | |  |
